# Assessing the Effect of Extreme Weather on Population Health Using Consumer-Grade Wearables in Rural Burkina Faso: Observational Panel Study

**DOI:** 10.2196/46980

**Published:** 2023-11-08

**Authors:** Mara Koch, Ina Matzke, Sophie Huhn, Ali Sié, Valentin Boudo, Guillaume Compaoré, Martina Anna Maggioni, Aditi Bunker, Till Bärnighausen, Peter Dambach, Sandra Barteit

**Affiliations:** 1 Heidelberg Institute of Global Health Faculty of Medicine, University Hospital Heidelberg University Heidelberg Germany; 2 Centre de Recherche en Santé Nouna Burkina Faso; 3 Charité - Universitätsmedizin Berlin, Institute of Physiology, Center for Space Medicine and Extreme Environments Berlin Berlin Germany; 4 Department of Biomedical Sciences for Health Università degli Studi di Milano Milan Italy; 5 Center for Climate, Health, and the Global Environment Harvard T.H. Chan School of Public Health Boston, MA United States; 6 Department of Global Health and Population Harvard T.H. Chan School of Public Health Boston, MA United States; 7 Africa Health Research Institute KwaZulu-Natal South Africa

**Keywords:** wearable, consumer-grade wearable, sleep, activity, heart rate, climate change, heat, rain, weather, sub-Saharan Africa, global health, public health, mobile phone

## Abstract

**Background:**

Extreme weather, including heat and extreme rainfall, is projected to increase owing to climate change, which can have adverse impacts on human health. In particular, rural populations in sub-Saharan Africa are at risk because of a high burden of climate-sensitive diseases and low adaptive capacities. However, there is a lack of data on the regions that are anticipated to be most exposed to climate change. Improved public health surveillance is essential for better decision-making and health prioritization and to identify risk groups and suitable adaptation measures. Digital technologies such as consumer-grade wearable devices (wearables) may generate objective measurements to guide data-driven decision-making.

**Objective:**

The main objective of this observational study was to examine the impact of weather exposure on population health in rural Burkina Faso using wearables. Specifically, this study aimed to assess the relationship between individual daily activity (steps), sleep duration, and heart rate (HR), as estimated by wearables, and exposure to heat and heavy rainfall.

**Methods:**

Overall, 143 participants from the Nouna health and demographic surveillance system in Burkina Faso wore the Withings Pulse HR wearable 24/7 for 11 months. We collected continuous weather data using 5 weather stations throughout the study region. The heat index and wet-bulb globe temperature (WBGT) were calculated as measures of heat. We used linear mixed-effects models to quantify the relationship between exposure to heat and rainfall and the wearable parameters. Participants kept activity journals and completed a questionnaire on their perception of and adaptation to heat and other weather exposure.

**Results:**

Sleep duration decreased significantly (*P*<.001) with higher heat exposure, with approximately 15 minutes shorter sleep duration during heat stress nights with a heat index value of ≥25 °C. Many participants (55/137, 40.1%) reported that heat affected them the most at night. During the day, most participants (133/137, 97.1%) engaged in outdoor physical work such as farming, housework, or fetching water. During the rainy season, when WBGT was highest, daily activity was highest and increased when the daily maximum WBGT surpassed 30 °C during the rainiest month. In the hottest month, daily activity decreased per degree increase in WBGT for values >30 °C. Nighttime HR showed no significant correlation with heat exposure. Daytime HR data were insufficient for analysis. We found no negative health impact associated with heavy rainfall. With increasing rainfall, sleep duration increased, average nightly HR decreased, and activity decreased.

**Conclusions:**

During the study period, participants were frequently exposed to heat and heavy rainfall. Heat was particularly associated with impaired sleep and daily activity. Essential tasks such as harvesting, fetching water, and caring for livestock expose this population to weather that likely has an adverse impact on their health. Further research is essential to guide interventions safeguarding vulnerable communities.

## Introduction

### Climate Change Effects on Human Health

There is growing scientific evidence that environmental conditions and extreme weather exposure associated with climate change are having negative effects on human health [1-3]. In particular, heat presents one of the most immediate health threats associated with climate change [2,4]. In combination with humidity, the health risks of heat can be exacerbated [5]. Previous studies have found that rising temperatures negatively affect sleep [6,7], which can cause, for example, decreased cognitive function [8], compromised immune function [9], and adverse cardiovascular outcomes [10]. Daily activity has also been found to be negatively affected by heat [11,12], which can cause reduced working capacity and productivity [13] in addition to adverse long-term effects on morbidity and mortality [14-16]. In rural populations in low-income countries, where people often rely on agriculture and livestock for their livelihood, higher exposure to extreme weather events may have adverse effects on nutrition and health. In addition, heart rate (HR) is another health parameter affected by heat. HR has been found to increase in hotter conditions [17,18], and increased HR is associated with numerous effects on the cardiovascular system [19].

### Climate Change and Health in Sub-Saharan Africa

Sub-Saharan Africa, including Burkina Faso, is expected to be severely affected by climate change [20]. The average surface temperature across Africa is projected to increase at a higher rate than the global average [21]. Changes in rainfall patterns are already causing severe droughts and floods [22]. Despite the mounting scientific evidence, population-level health effects of climate change in Africa, especially in low-resource contexts in the sub-Saharan region, are still poorly understood because of a lack of objective measurements of health parameters in response to exposure to extreme weather events [2,23]. African populations are especially at risk because of a high burden of climate-sensitive diseases and low adaptive capacity [22,24].

### Research Infrastructures in Low- and Middle-Income Countries

To inform public health actions and identify emerging health concerns and increased-risk groups, public health surveillance is crucial [25,26]. In general, there is a scarcity of continuous and spatially distributed data available in low- and middle-income countries, particularly in sub-Saharan Africa, to conduct research on the effects of climate change on population health. To that end, research infrastructures such as health and demographic surveillance systems (HDSSs), of which >50 have been implemented across Asia and Africa, are important ecosystems capable of surveilling geographically defined populations. HDSSs provide valid and reliable population-based data on population dynamics (birth, death, and in- and out-migration), particularly in areas with inadequate or nonexistent vital event registration and health information systems. The Nouna HDSS, located in northwestern Burkina Faso, has been collecting long-term data on the health and demographics of a population of >120,000 individuals since 1992 [27].

Previous research on climate change and health in the Nouna HDSS has shown the relationship between climate variations and nutritional outcomes in children aged <5 years [28] and the impact of varying climate and weather conditions on population mortality, with a high indication of excess burden of noncommunicable disease and mortality [29,30]. However, most HDSSs do not capture local-level weather parameters such as temperature and precipitation, which are particularly key indicators of exposure to extreme weather events [22]. They are also unable to produce more comprehensive heat measures such as the heat index (HI) or wet-bulb globe temperature (WBGT), which have been found to be better indexes for heat exposure than temperature alone [31,32]. HDSSs can also provide continuous real-world surveillance of an individual’s health by obtaining objective measurements and highly resolved health data if new sensors such as consumer-grade wearable electronic devices (hereinafter referred to as *wearables*) are incorporated [33].

### Consumer-Grade Wearables for Climate Change and Health Research

Consumer-grade wearables, including the Withings Pulse HR, were introduced for the first time in the Nouna HDSS and were found to be a feasible and acceptable method for continuous health surveillance of individuals, allowing for ecological momentary assessments [34]. A number of studies have used wearables in climate change and health research, but none have used wearables in low-income countries to assess the health effects of extreme weather [35]. To our knowledge, there is no population-wide effort to systematically monitor populations at an individual level to better quantify and characterize the effects of climate change on health. Data on the daily effects of climate change on people’s lives and health are currently scarce and are crucial to tailor interventions and adaptation measures to vulnerable populations. Climate change adaptation is essential to protect vulnerable groups in low-resource environments from rising average temperatures and exposure to extreme weather.

The overarching objective of this observational study was to examine the impact of heat and heavy rainfall on the population’s health in rural Burkina Faso using consumer-grade wearables. Specifically, we captured daily activity, sleep, and HR using a wearable in a sample of the Nouna HDSS population. Our primary objectives were to study the relationships between (1) daily activity and heat and heavy rain, (2) nighttime sleep duration and heat and heavy rain, and (3) HR and heat and heavy rain.

Our secondary research question focused on the stratification of these relationships according to month and different demographic subgroups, specifically for sex, age group, and BMI group.

## Methods

### Study Design

We conducted an observational panel study covering a population of 143 participants in the Nouna HDSS in northwestern Burkina Faso from August 2021 to June 2022. During the 11 months of data collection, study participants were equipped with wearables (Withings Pulse HR) that they wore continuously. Weather data were collected at 5 weather stations throughout the study area. This study is reported according to the STROBE (Strengthening the Reporting of Observational Studies in Epidemiology) statement: guidelines for reporting observational studies (Multimedia Appendix 1).

### Study Setting and Population

The study was conducted in the Nouna HDSS in northwestern Burkina Faso, which is located approximately 40 km from the Mali border and 250 km from the capital, Ouagadougou. The region’s tropical climate is defined by one rainy season, which typically lasts from June to September, and high temperatures throughout the year [36].

Individuals were eligible for study participation if they (1) were aged ≥16 years, (2) had no plans for long-term travel during the study period, and (3) consented to study participation.

We calculated a sample size of 150 participants based on an eligible population of 100,000 (total HDSS population aged >6 years), a confidence level of 95%, and an error margin of 8%. To ensure that each sex was equally represented in the study population, random sampling was stratified by sex (for details on the sampling, see the study protocol by Barteit et al [37]).

### Ethics Approval

Ethics approval was granted by the Comité d’ethique pour la recherche en santé in Burkina Faso (approval date: March 13, 2020; 2020-3-041) and by the ethical committee of the Heidelberg University Hospital, Germany (approval date: May 6, 2019; S-294/2019).

### Study Proceedings

#### Weather Data

**Figure 1:**
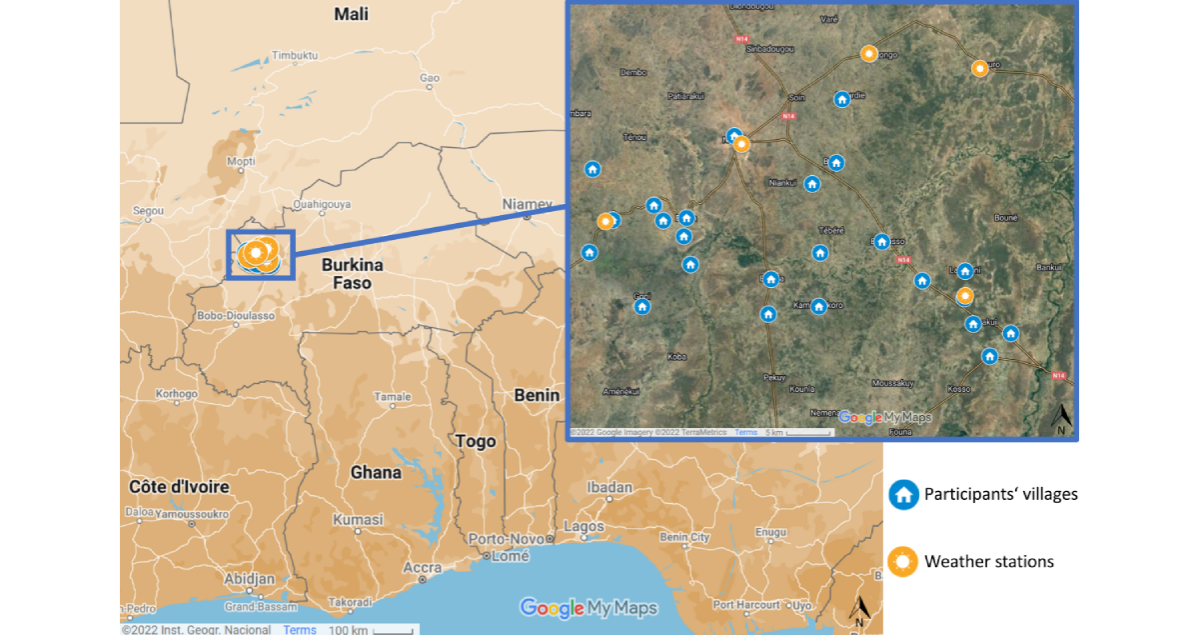
Spatial distribution of weather stations across the Nouna health and demographic surveillance system area (image on the left) and the study area with the weather stations and the respective villages of participants involved in the study (image on the right). Map data: Google, Inst. Geogr. Nacional, created with Google MyMaps 2022.

In mid-2020, a total of 5 weather stations were set up to cover the spatial variability of different weather exposures across the study area. The nearest weather station was assigned to each of the 25 study villages (Figure 1). The distance was calculated as the shortest distance between 2 points on a WGS 84 ellipsoid using the distGeo function of the R package *geosphere* (version 1.5.18 [38]; R Foundation for Statistical Computing), which accounts for the ellipsoid shape of the Earth’s surface.

#### Consumer-Grade Wearables

**Figure 2:**
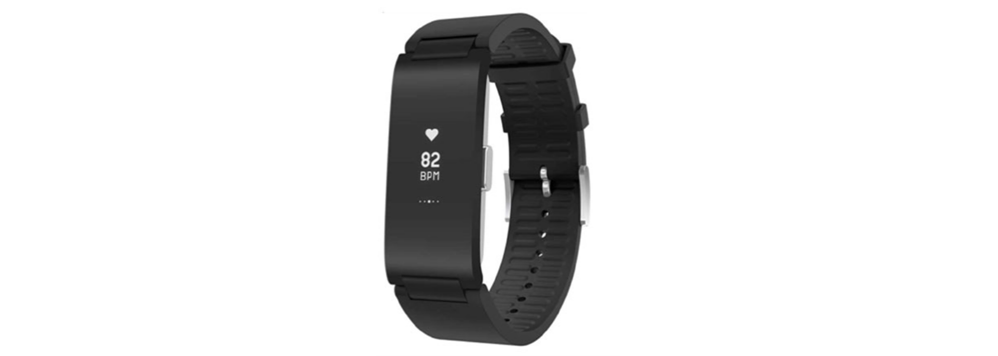
Withings Pulse HR consumer-grade wearable that participants wore during the study.

From August 2021 to June 2022, study participants were provided with consumer-grade wearable devices (Withings Pulse HR; Figure 2) and instructions on how to wear them correctly on their wrists (correct position and tightness). Participants wore the Withings Pulse HR for the entire study period. Considering that electricity is not available in all households in the study area, participants received a foldable solar panel with USB ports to charge the wearable and a smartphone to synchronize its data (for details, see the study protocol by Barteit et al [37]). Participants received weekly visits from a fieldworker. At each visit, the fieldworker checked the functionality of the wearable and smartphone, charged all devices with a portable power bank, and synchronized the data to the server for remote data web access. Wearables that were damaged during the study were replaced.

#### Activity Journals and Heat Questionnaires

The 21 most prevalent local activities were listed in a structured activity journal that study participants completed once a month according to their activities after getting up, in the morning, at noon, in the afternoon, in the evening, and at night (Multimedia Appendix 2). At the end of the study period, a questionnaire was administered evaluating the perception of heat with multiple-choice questions regarding whether, when, and how heat affected participants and their adaptation strategies (Multimedia Appendix 2).

### Technical Measurement Details

#### Weather Stations

The weather stations were equipped with ADCON sensors and measured several variables, including air temperature, relative humidity, precipitation, wind speed and direction, and global radiation, at 15-minute intervals. The data were uploaded using the advantage Pro software (version 6.8; ADCON telemetry, OTT Hydromet GmbH).

#### Consumer-Grade Wearables

Consumer-grade wearables are waterproof and feature a 3-axis accelerometer that continuously estimates steps (identified by amplitude and periodic pattern) and sleep parameters (duration, time spent awake, sleep onset and offset, and sleep depth) through automatic data postprocessing using a manufacturer’s algorithm. Steps have been frequently used in research to quantify daily activity [16]. Sleep duration specifies the total recorded time that a study participant spent asleep on a given night. Sleep interruptions were defined as wake time after sleep onset, whereas sleep onset was defined as the time of falling asleep and sleep offset was defined as the time of waking up. In addition, the device’s photoplethysmography sensor estimates the pulse rate every 10 minutes or every 90 seconds in activity mode. For the purpose of this study, we deemed the pulse rate and HR to be equivalent. One charging cycle can last up to 21 days, and the internal storage can hold up to 5 days of recorded data. The data collected from the wearables in this study were wirelessly transmitted via Bluetooth to an app on the study participants’ smartphone and, when an internet connection was available, uploaded from the mobile device to the Withings server (*health mate*).

### Data Processing

#### Demographic Data

Data on weight, height, and date of birth and death were available for each study participant. Participants were categorized as young adults (aged <25 years), middle-aged adults (aged ≥25 and <65 years), and older adults (aged ≥65 years) based on their age at the beginning of the study period. BMI was calculated as weight (kg)/(height (m) × height (m) and grouped into 3 categories: underweight (<18.5 kg/m^2^), healthy weight (≥18.5 and <25 kg/m^2^), and overweight (≥25 kg/m^2^).

#### Weather Data

We calculated WBGT estimates according to the formula provided by Carter et al [39] and used the heat.index function of the R package *weathermetrics* (version 1.2.2 [40]) to calculate the HI according to the US National Weather Service complex algorithm from temperature and relative humidity [40].

Weather extreme indexes were calculated for each day of the study period so that days with and without extreme weather could be compared. As weather extremes are often characterized relative to historical data (90th percentile of a 30-year reference period) [41] and we only had approximately 2 years of weather data from the 5 weather stations that were installed in the Nouna HDSS in 2020, we used the following climate extreme indexes developed by Climpact and recommended by the World Meteorological Organization Expert Team on Sector-specific Climate Indices: (1) number of days with heavy precipitation (count of days in which the daily precipitation was ≥20 mm), (2) number of tropical nights (count of nights in which the minimum temperature was >20 °C), and (3) number of hot days (count of days in which the daily maximum temperature was ≥35 °C).

In addition, we calculated weather extreme indexes based on WBGT and HI (also called apparent temperature) to provide a more accurate assessment of heat exposure [31,32]. We used WBGT as a heat parameter during the daytime and HI during the nighttime. The WBGT calculation includes global radiation and wind speed, and as the data are measured using weather stations outdoors, we found HI to be more applicable than WBGT for the assessment of heat exposure during the night when participants were mostly indoors. On the basis of findings of current climate and health research using WBGT [7,42,43] and the National Weather Service cutoffs for HI [44], we used the following cutoffs: (1) the number of heat stress days was defined as the count of days in which the daily maximum WBGT was ≥30 °C, and (2) the number of heat stress nights was defined as the count of nights in which the minimum HI was ≥25 °C.

For correlating weather data with wearable data, we divided the weather data into daytime and nighttime data. On the basis of median sleep onset and offset times (median sleep onset 10:09 PM; median sleep offset 5:55 AM), cutoffs for nighttime weather were determined, resulting in measurements between 10 PM and 6 AM the following day. Daytime weather was defined as measurements between 6 AM and 10 PM. For comparison between seasons, weather data were also categorized into rainy (June-September), cool dry (October-January), or hot dry (February-May) season according to the month.

#### Consumer-Grade Wearables

##### Data Completeness

To increase the representativeness of the measurements and address missing wearable data, we considered participants who had data coverage of 25% of the measurement days as complete cases, similar to the study by Minor et al [6]. Using a 50% criterion yielded comparable results (Multimedia Appendix 3); however, HR data had too little completeness for a higher cutoff. We did not impute data as data imputation was found not to be necessary for unbiased results when only the outcome variable was affected by missing data [45]. In addition, studies have found that multiple imputation did not increase precision when using linear mixed models for data analysis [46]. Furthermore, Jakobsen et al [47] did not recommend using data imputation for a high percentage of missing data.

##### Daily Activity

In line with previous studies, a filter of at least 10 hours of wear time per participant per day was used to exclude days with insufficient wear time [12,48,49]. We defined nonwear time as more than 1 hour between measurements. Values of 0 steps were excluded as, according to the manufacturer, nonwear time and a measurement of 0 steps cannot be distinguished. Duplicate measurements were removed. We aggregated the steps into 15-minute intervals to be consistent with weather data intervals and also as daily step counts as a measure of daily activity.

##### Sleep Data

We limited sleep length based on the onset and offset times to eliminate incorrect values. Adapted from the study by Minor et al [6], we defined nighttime sleep with a sleep onset of ≥5 PM and a sleep offset of ≤1 PM as limits, with a 2-hour adjustment to account for earlier bedtimes in this study population. If a participant had multiple sleep observations in a single night, they were summarized into one sleep observation by adding the sleep duration values and the duration between the first offset and second onset to wake time after sleep onset. If multiple sleep observations overlapped for one participant, they were excluded as error measurements. In accordance with the manufacturer’s declaration that sleep detection measurements with a time difference of <3 hours between sleep onset and sleep offset are invalid measurements, we excluded those measurements.

##### HR Measurement

Duplicate HR measurements were removed, and values greater than the age-predicted maximal HR according to the equation by Tanaka et al [50] (208 – 0.7 × age) were excluded. HR measurements were rounded and aggregated (mean, minimum, and maximum) into 15-minute intervals to be consistent with weather data intervals. HR measurements were divided into daytime and nighttime HRs using the median sleep onset and offset as nighttime definitions (10 PM-6 AM). Measurements for 1 day were included when at least 2 hours of measurements were available; nighttime measurements for 1 night were included when at least 1 hour was covered by at least one measurement every 15 minutes. We chose the threshold of 15 minutes in accordance with previous research [34].

### Statistical Analysis and Data Modeling

#### Descriptive Data Analysis

All data analyses were conducted using R (RStudio version 2022.07.02+576; Posit, PBC).

First, the weather and wearable data were descriptively analyzed (covering minimum, maximum, mean, and SD values). Activities reported in the activity diaries were thematically summarized into 9 categories and quantified relative to the total number of activity journal responses. Similarly, the heat questionnaire was analyzed, and responses were summarized based on the frequency of replies.

#### Mixed-Effects Models

##### Associations Between Weather Exposure and Daily Activity (Steps), Sleep Duration, and Nighttime HR

We conducted a linear mixed-effects analysis of the relationship between weather exposure and daily activity (steps), sleep duration, and nighttime HR using the lmer function of the *lme4* R package (version 1.1.34 [51]). We used the following formula:

Y_i_ = b_0_ + b_1_Heat_i_ + b_2_Precipitation_i_ + b_z_Z_i_ + ε_i_ **(1)**

In this linear mixed-effects model, *i* indicates each study participant. The dependent variable *Y_i_* sequentially represents sleep duration (in hours), daily activity (in steps), and average nighttime HR (in beats per minute [bpm]) of individual *i*. The independent variable of interest “Heat” represents the minimum nighttime HI (HI_min_) for sleep and nighttime HR and maximum daytime WBGT (WBGT_max_) for daily activity (steps). The independent variable “Precipitation” represents the total daily rainfall. Furthermore, we controlled for month, weekend or weekday, age group, sex, and BMI group by adding these variables stepwise as independent variables, represented as *Z*. Weekend was added as a possible confounder as we expected the participants’ activities to vary between weekends and weekdays, also affecting nighttime sleep. Similarly, agricultural activity most likely varies between months. Furthermore, by adding month as an independent variable, we indirectly also accounted for differences in daylight hours. The demographic confounders sex, age, and BMI group have frequently been shown to be associated with sleep, HR, and daily activity and, therefore, were included in our models.

By adding study participants as a random term, the linear mixed-effects model accounted for the nonindependence of the repeated measures. In addition, linear mixed models were chosen as they can estimate parameters from existing data to handle missing data and independent variables can be on a continuous scale with differing times between measurement points. We used maximum likelihood for the estimation of the model parameters. In total, 3 separate models were constructed for sleep duration, daily activity, and average nighttime HR. Covariates were added through hierarchical model building, with removal based on the chi-square likelihood test (lower log-likelihood values were removed) and a significance level of 5%. We used leave-one-subject-out (LOSO) cross-validation to assess model performance. We compared the performance of the resulting models on the respective validation sets using *R*^2^ and root mean square error as well as the average values for these 2 parameters.

If visual inspection of the residual plots revealed deviation from linearity, we introduced a quadratic term for the predictor variable using the following equation:

Y_i_ = b_0_ + b_1_Heat_i_ + b_2_Heat^2^_i_ + b_3_Precipitation_i_ + b_z_Z_i_ + ε_i_ **(2)**

We created an additional model for each of the 3 wearable parameters using temperature and daily rainfall as predictors to account for heat metrics other than WBGT and HI. Model selection was based on the Akaike information criterion, with a lower value indicating an improved model fit.

##### Differences in Subgroup Sensitivity to Heat

We evaluated the effect of heat exposure on daily activity, sleep duration, and nighttime HR between the different subgroups. This way, it was possible to assess the different heat sensitivity of each subgroup. Therefore, we included an interaction term between continuous measures for heat and a categorical variable *X*, which successively represented age group, BMI group, sex, or month. If adding the interaction term significantly improved the model, we ran the model for each subset separately to compare the effects of heat exposure. We interpreted the results for each subgroup relative to its corresponding reference category (middle-aged adults, healthy weight participants, male individuals, and coldest month).

Y_i_ = b_0_ + b_1_Heat_i_ × X + b_2_Precipitation_i_ + b_z_Z_i_ + ε_i_ **(3)**

##### Associations Between Weather Extremes and Daily Activity, Sleep Duration, and Nighttime HR

We built linear mixed-effects models using binary variables for heat (heat stress day or night) and heavy rainfall as independent variables in place of the continuous variables to evaluate the association between weather extremes and the wearable parameters. The model formula changed as follows:

Y_i_ = b_0_ + b_1_Heatstress_i_ + b_2_HeavyPrecipitation_i_ + b_z_Z_i_ + ε_i_ **(4)**

## Results

### Participant Characteristics

We originally recruited 152 participants. During the study, of these 152 participants, 7 (4.6%) withdrew their consent, 1 (0.7%) died, and 1 (0.7%) migrated out of the study region. Therefore, the results report on a total of 143 study participants.

Approximately half (71/143, 49.7%) of the study population was female. The age of the study participants ranged from 16 to 79 years, with an average age of 43 (SD 13) years. Most (128/143, 89.5%) were middle-aged (aged 25-65 years). The average BMI of the participants was 22.3 (SD 2.7) kg/m^2^, ranging from 16.6 to 32.42 kg/m^2^, with most (112/143, 78.3%) falling into the healthy weight category (18.5≤BMI<25).

**Figure 3:**
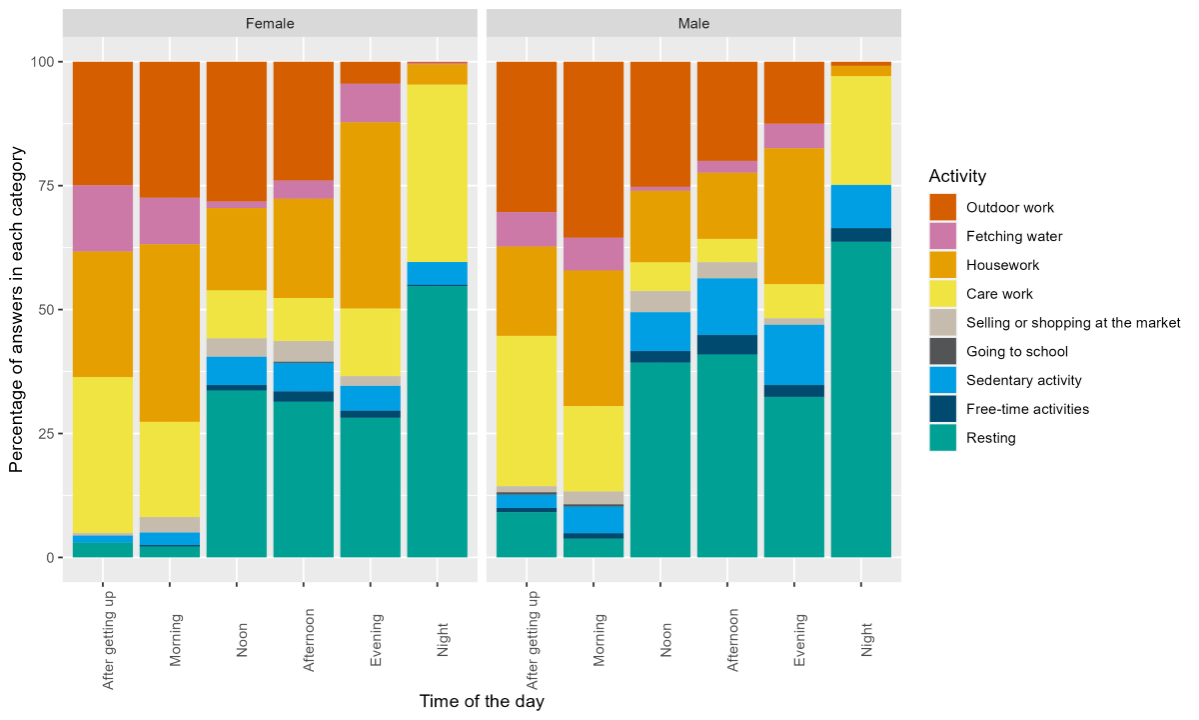
Self-reported daily activities of study participants stratified by sex and time of day (after getting up, in the morning, at noon, in the afternoon, in the evening, and at night).

When looking at the distribution of self-reported activities throughout the day (Figure 3), we observed that after waking up and in the morning were the most active times of the day, when participants engaged in outdoor work, housework, and errands such as fetching water and care work. The rest of the day was often spent resting. Male participants reported more outdoor work, whereas female participants reported more care work and housework, especially in the evening and at night.

### Weather

The average distance between the villages and the closest weather station was 7.6 km (range 0.1-19.0, SD 14.4 km). For the entire study region (average of 4 weather stations), the temperature range was 10.9 to 44.0 °C with a mean of 28.5 °C, and WBGT ranged from 7.3 to 32.8 °C with a mean of 23.3 °C. The maximum daily precipitation measured was 101.5 mm with an average of 1.9 mm per day and an average cumulative sum over the entire study period of 630.5 mm. During the 1-year study period, there were, on average, 121 (range 110-131) days classified as heat stress days with maximum WBGT values of >30 °C and an average of 117 (range 102-135) nights classified as heat stress nights with minimum HI values of >25 °C. Furthermore, study participants were exposed to heavy rainfall, with ≥20 mm on average on 8 (range 6-11) study days and single measurements exceeding 50 mm of precipitation per day on 7 study days. Table 1 provides a detailed overview of weather data stratified by season.

**Table 1:** Weather parameters and extreme weather events for the study duration of 11 months by season (average of all 4 closest weather stations).

Weather exposure	Rainy season (June-September)	Cool dry season (October-January)	Hot dry season (February-May)
Mean air temperature (°C; SD)	27.6 (3.8)	26.8 (6.4)	31.1 (6.0)
Mean WBGT^a^ estimate (°C; SD)	25.9 (2.5)	20.5 (5.6)	23.4 (4.9)
Number of days with maximum daily air temperature of ≥35 °C	24	73	104
Number of days with maximum daily WBGT estimate of ≥30 °C	46	33	42
Number of days with rainfall of ≥20 mm	8	0	1
Number of nights^b^ with minimum air temperature of ≥20 °C	91	48	98
Number of nights^b^ with minimum HI^c^ of ≥25 °C	40	15	63

^a^WBGT: wet-bulb globe temperature.

^b^Nights were defined as 10 PM to 6 AM (median sleep onset–median sleep offset).

^c^HI: heat index.

### Wearable Data

#### Data Completeness

**Figure 4:**
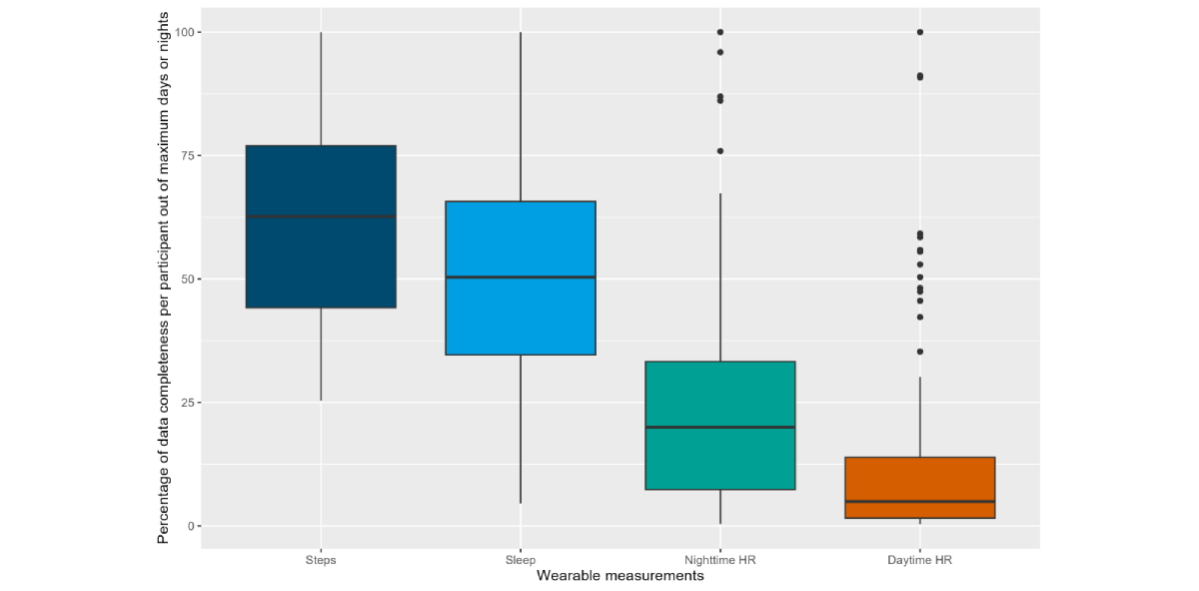
Box plot of data completeness per wearable parameter in percentage of the respective maximum number of days or nights with data for 1 participant. HR: heart rate.

The data completeness of wearable measurements of daily activity, sleep, and HR is shown in Figure 4. The average data completeness per participant was 166 (SD 66) days for daily activity; 153 (SD 69) nights for sleep; and 58 (SD 51) nights and 34 (SD 51) days for nighttime and daytime HR, respectively.

#### Daily Activity Measurements

##### Overview

**Figure 5:**
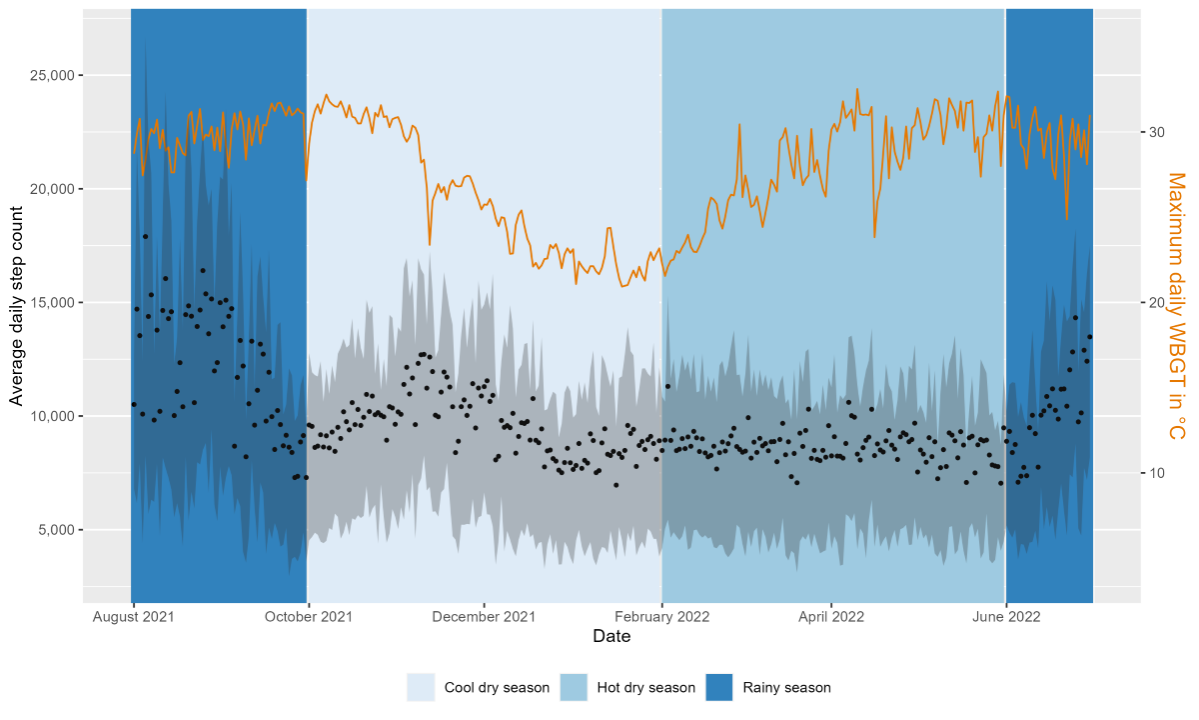
Average daily steps (black dots) with IQR (gray area) and maximum daily wet-bulb globe temperature (WBGT; °C; orange line) for the duration of the study. The background color indicates the 3 seasons (cool dry, hot dry, and rainy).

We had to remove 26.64% (2,438,464/9,152,256) of the data points, with the final data set including a total of 6,713,792 step measurements and 22,853 days from 90.9% (130/143) of the participants. The mean daily activity (in steps) was 9602 (SD 6499; minimum: 194; maximum: 47,522). Figure 5 shows that the average number of steps taken each day changed based on the season. In the rainy season, the average number of steps was 11,328 (SD 8049); in the cool dry season, it was 9532 (SD 6140); and, in the hot dry season, it was 8700 (SD 5681).

**Figure 6:**
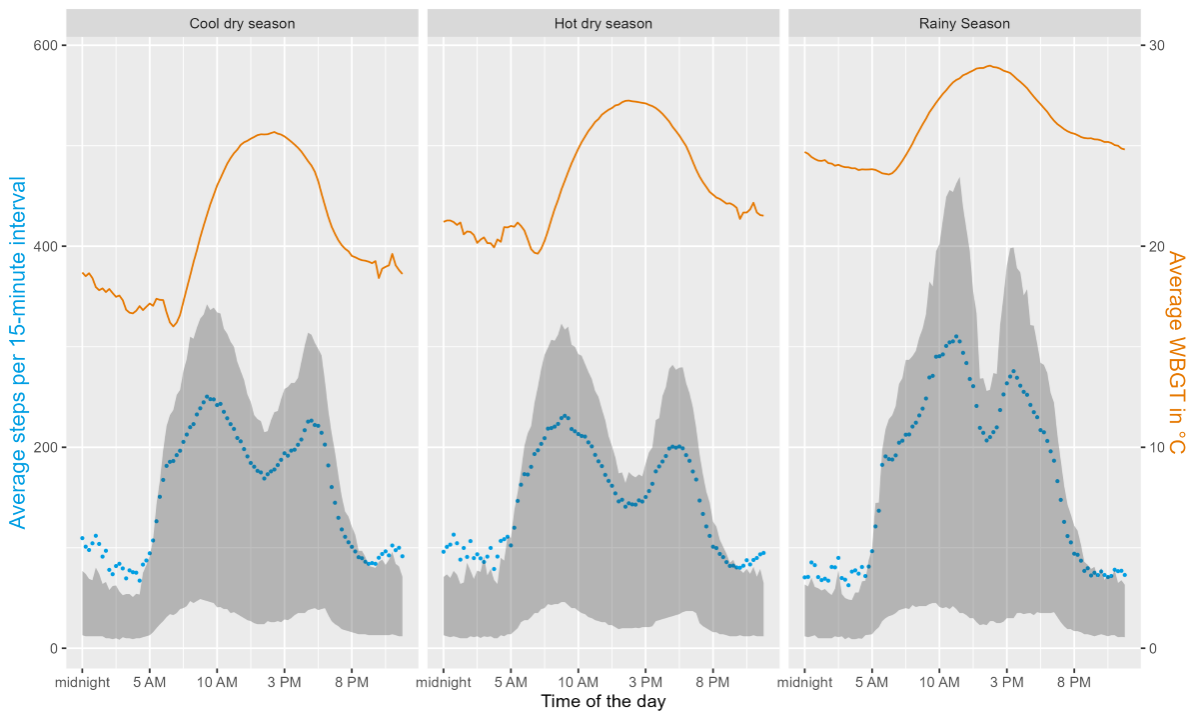
The distribution of the average number of steps per 15-minute interval (blue dots) with the IQR (gray area) over the course of a day and wet-bulb globe temperature (WBGT; measured every 15 minutes; orange line) for the 3 seasons in Burkina Faso (cool dry, hot dry, and rainy).

The distribution of steps over the course of the day also varied from season to season (Figure 6). There were activity peaks in the morning and late afternoon during all seasons. In the rainy season, the peak occurred at 11:15 AM with a mean of 310 (SD 367) steps per 15-minute interval, whereas in the hot dry season, it occurred at 9 AM with a mean of 231 (SD 269) steps per 15-minute interval.

##### Weather Exposure and Daily Activity Measurements (Steps)

We fitted a linear mixed model with participants as a random effect. Daily steps showed significant variance in intercepts across participants (χ^2^_1_=9455.8; *P*<.001). Maximum daily WBGT (WBGT_max_) and total daily precipitation were included as predictors. WBGT_max_ as a fixed effect was highly significant (2-tailed *t* test, *t*_22,849_=12.73; *P*<.001). However, as the residual plot of daily activity and WBGT_max_ showed nonlinearity, we added a quadratic term for WBGT_max_ (WBGT_max_^2^), which improved the model fit. Herein, we report the combined effects of WBGT_max_ and WBGT_max_^2^. For a WBGT_max_ of 20 °C, we found an increase of 146 steps for every additional degree of WBGT (°C), whereas for a WBGT_max_ of 30 °C, we observed a decrease of 83 steps for every additional degree of WBGT (°C). The main effect of daily precipitation was also significant (*t*_22,725_=−6.57; *P*<.001), and we found that daily activity decreased by an average of 39 steps for every 1-mm increase in total daily rainfall (95% CI −44 to −23). The final model included adjustments for age group, month, and weekend or weekday. We also developed a model using the maximum daily temperature in °C as a predictor instead of WBGT_max_, which was shown to be a less accurate model. We cross-validated the model using the LOSO approach and calculated *R*^2^ values of 0.45 for some participants as test data, with an average of 0.12.

##### Different Heat Sensitivity by Age, BMI, Sex, and Month

To assess the correlation between different subgroups and the effect of WBGT_max_ on daily activity, we added an interaction term for age group, sex, or month and WBGT_max_ and WBGT_max_^2^. If the addition of the interaction term improved the fit of the model, we ran the model independently for each subgroup to compare the effect of WBGT_max_ on daily activity. Table 2 provides a summary of the per-degree effects of WBGT_max_ on daily activity for each subgroup. For different sex and age groups, we did not find significant differences in heat sensitivity (*P*>.05). During the hottest month (April) and the coolest month (January), the effect of increasing WBGT_max_ on daily activity was not significant. During August, the rainiest month, the effect of increasing WBGT_max_ on daily activity was opposite to that during January and April, with a 5126-step decrease per degree increase in WBGT_max_ at 20 °C (compared with an increase in January and April) and an increase of 678 steps (compared with a decrease in January and April) at 30 °C.

**Table 2:** Combined effect of 1-degree increases in maximum wet-bulb globe temperature (WBGTmax) and squared WBGTmax (WBGTmax2) on daily activity (in steps) for different age groups and sex and during different seasons.

Subgroup	Combined effect of WBGT_max_ and WBGT_max_^2^ at 20 °C on steps	Combined effect of WBGT_max_ and WBGT_max_^2^ at 30 °C on steps	Estimate for WBGT_max_	*P* value for WBGT_max_	Estimate for WBGT_max_^2^	*P* value for WBGT_max_^2^
All participants (n=130)	+146	−83	606	.03	−12	.02
Older adults (aged ≥65 years; n=9)	+118	−60	475	.38	−9	.37
Middle-aged adults (aged 25-65 years; n=114)	+151	−93	638	.03	−12	.02
Young adults (aged <25 years; n=7)	+207	−10	641	.94	−11	.61
Male individuals (n=65)	+142	−88	601	.13	−11	.11
Female individuals (n=65)	+138	−67	547	.14	−10	.13
Rainiest month (August; n=79)	−5126	678	−16,734	.03	290	.02
Coolest month (January; n=122)	+370.34	−1047.06	3205.14	.35	−71	.36
Hottest month (April; n=99)	+304	−18	965	.43	−16	.45

##### Weather Extremes and Daily Activity

We further assessed the differences in daily activity between exposure to weather extremes (heat stress days and heavy rainfall) and nonexposure. We created a linear mixed model with the weather extreme exposure as binary fixed effects instead of WBGT_max_ and total daily rainfall. The final model was adjusted for age group, month, and weekend or weekday. We found no statistically significant difference in daily activity on heat stress days with WBGT_max_ ≥30 °C compared with days with WBGT_max_ <30 °C (*t*_22,725_=−0.64; *P*=.52). On days with heavy rainfall compared with days without heavy rainfall (*t*_22,724_=−3.25; *P*<.001), daily activity was significantly lower, with an estimate of −855 steps. The results for all 3 models can be found in Multimedia Appendix 3.

#### Sleep Measurements

##### Overview

**Figure 7:**
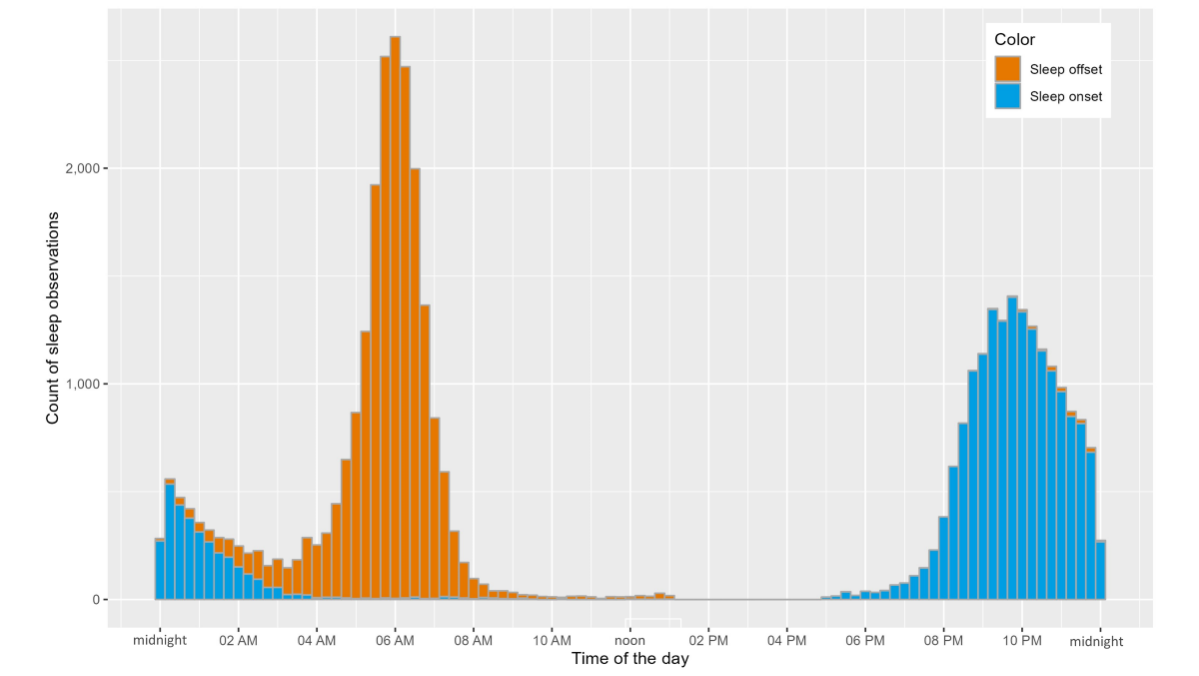
Histogram of the time that study participants fell asleep (orange colored) at night and woke up (blue colored) in the morning.

**Figure 8:**
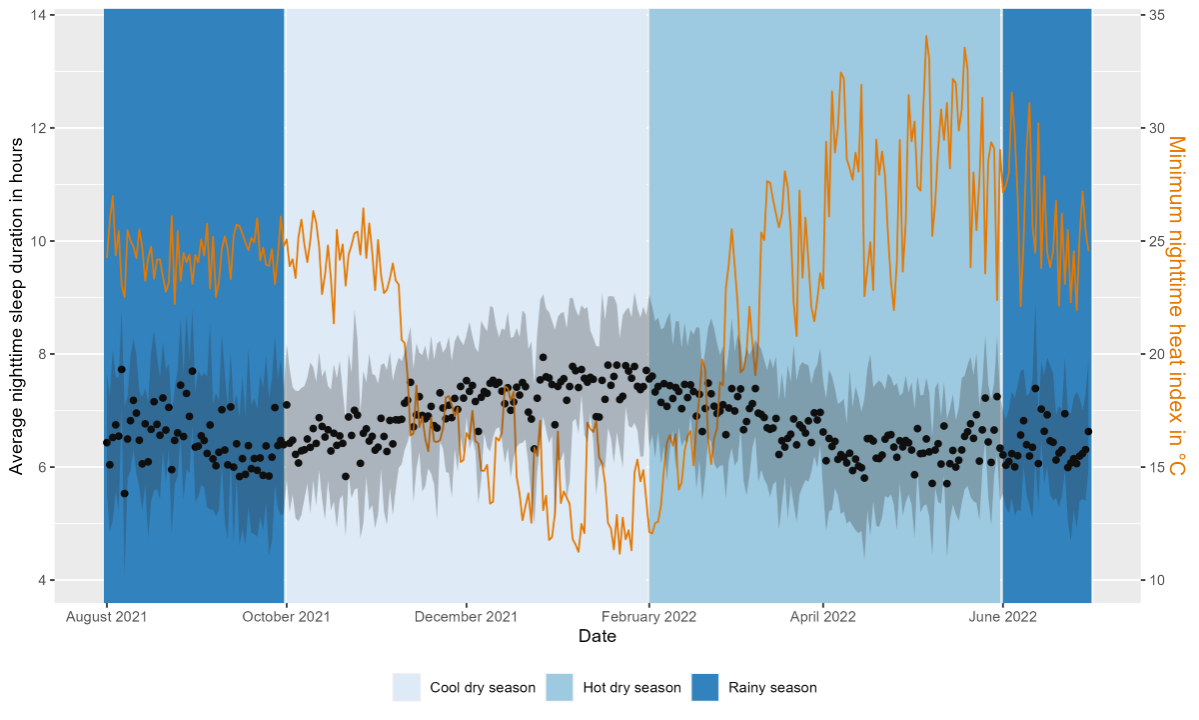
Average nighttime sleep duration (hours; black dots) with the IQR (gray area) and minimum heat index (°C; orange line; average of all 4 weather stations) throughout the study period. The background color indicates the 3 seasons (cool dry, hot dry, and rainy).

After removing 6.89% (1523/22,095) of the sleep observations during data processing, the final data set comprised 20,572 nights covering the study duration of 334 nights from 83.2% (119/143) of the study participants. The average sleep duration was 6 hours, 49 minutes (SD 1 min, 48 s) per night, the time of sleep onset was 10:23 PM (SD 1 h, 47 min), and the time of sleep offset was 05:44 AM (SD 1 h, 28 min; Figure 7). On average, study participants were awake for 36 (SD 30) minutes after sleep onset. Nearly half (10,012/20,505, 48.83%) of the participant nights showed insufficient sleep according to the age group definitions of sufficient sleep duration by Hirshkowitz et al [52]. This definition states that sleep duration of <8 hours for those aged <18 years and <7 hours for those aged >18 years is insufficient. The average sleep duration was shorter throughout the study when the minimum nighttime HI was higher (Figure 8).

##### Weather Exposure and Sleep Measurements (Duration)

To provide a detailed analysis of the relationship between sleep duration and nighttime heat, we fitted a linear mixed-effects model with study participants as a random term and the minimum nighttime HI (HI_min_) as a predictor of sleep duration. Sleep duration showed significant variance in intercepts across participants (χ^2^_1_=3596.9; *P*<.001). HI_min_ as a fixed effect was highly significant (*t*_20,520_=−9.60; *P*<.001). For every 1-degree increase in HI_min_, sleep duration decreased on average by 0.04 hours (2.4 minutes; 95% CI −0.074 to −0.065) in the fully adjusted model. We also included total daily rainfall as a predictor and found a significant (*t*_20,457_=6.27; *P*<.001) but small main effect of an increase of 0.01 hours (36 seconds; 95% CI 0.007-0.013) in sleep duration per mm increase in rainfall. The final model included adjustments for age group, BMI group, month, and weekend or weekday. Sex was considered as a covariate but was removed after failing to reach statistical significance (*P*<.05). We cross-validated the model using the LOSO approach and calculated *R*^2^ values of 0.34 for some participants as test data, with an average of 0.10. In addition, we developed a model using the minimum nighttime temperature in °C as a predictor of HI_min_ instead, which we found to be less accurate.

##### Different Heat Sensitivity by Age, BMI, Sex, and Month

By sequentially adding an interaction term for age group, BMI group, sex, and month with HI_min_, we assessed whether these subgroups exhibited varying levels of heat impact regarding sleep duration. We found no statistically significant differences between age groups, BMI groups, or sex. In the hottest month (April), the impact of heat was lowest, with an estimate for the effect of HI_min_ of −0.02 (SE 0.01) on sleep duration. In the coolest and driest month (January), the estimate for the effect of HI_min_ was −0.04 (SE 0.02), and in the rainiest month (August), the impact of heat was highest, with an estimate of −0.125 (SE 0.04).

##### Weather Extremes and Sleep Duration

We further assessed the difference in sleep duration between exposure to nights with heat stress and heavy rainfall and nonexposure. We created a linear mixed model with the binary weather extreme factors as fixed effects. Adding heavy rainfall as a fixed effect did not improve the model fit. The final model was adjusted for month and weekend or weekday. We found significantly (*t*_20,470_=−7.81; *P*<.001) shorter sleep duration on heat stress nights with HI_min_ ≥25 °C compared with nights with HI_min_ <25 °C by an estimated 0.24 hours (15 minutes; 95% CI −0.31 to −0.18). The results for all 3 models of sleep duration can be found in Multimedia Appendix 3.

#### HR Measurements

##### Overview

The data-cleaning process removed 88.55% (488,906/552,099) of the HR measurements. We split the measurements into daytime and nighttime HR. The final data set of daytime HR comprised a total of 3062 fifteen-minute intervals collected across 249 participant days from 11.9% (17/143) of the study participants spanning 190 study days. Nighttime HR data included 31,423 fifteen-minute intervals from 32.2% (46/143) of the participants collected across 3025 participant days.

During the day, the average HR was 90 (SD 23) bpm; during the night, the average HR was 69 (SD 13) bpm. We further only considered nighttime HR as an outcome variable as daytime measures had low data completeness for HR.

**Figure 9:**
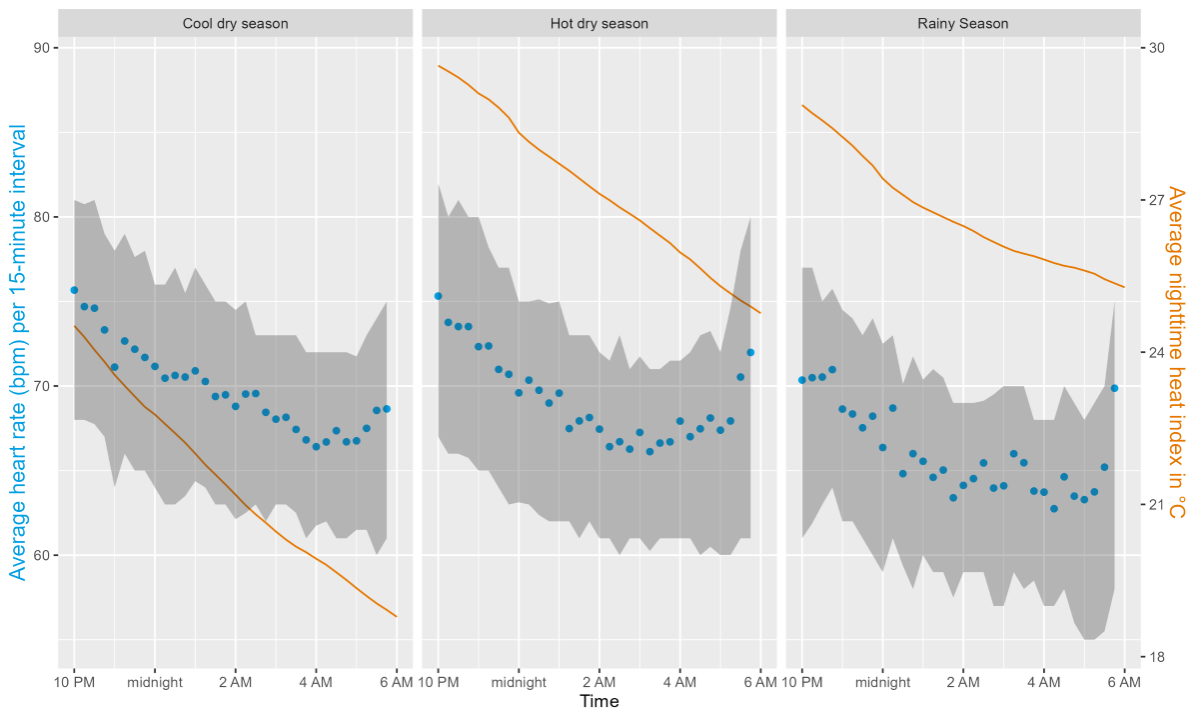
Average heart rate in beats per minute (bpm; blue dots) with the IQR (gray area) per 15-minute interval and average heat index (orange line) in °C during the night by season.

**Figure 10:**
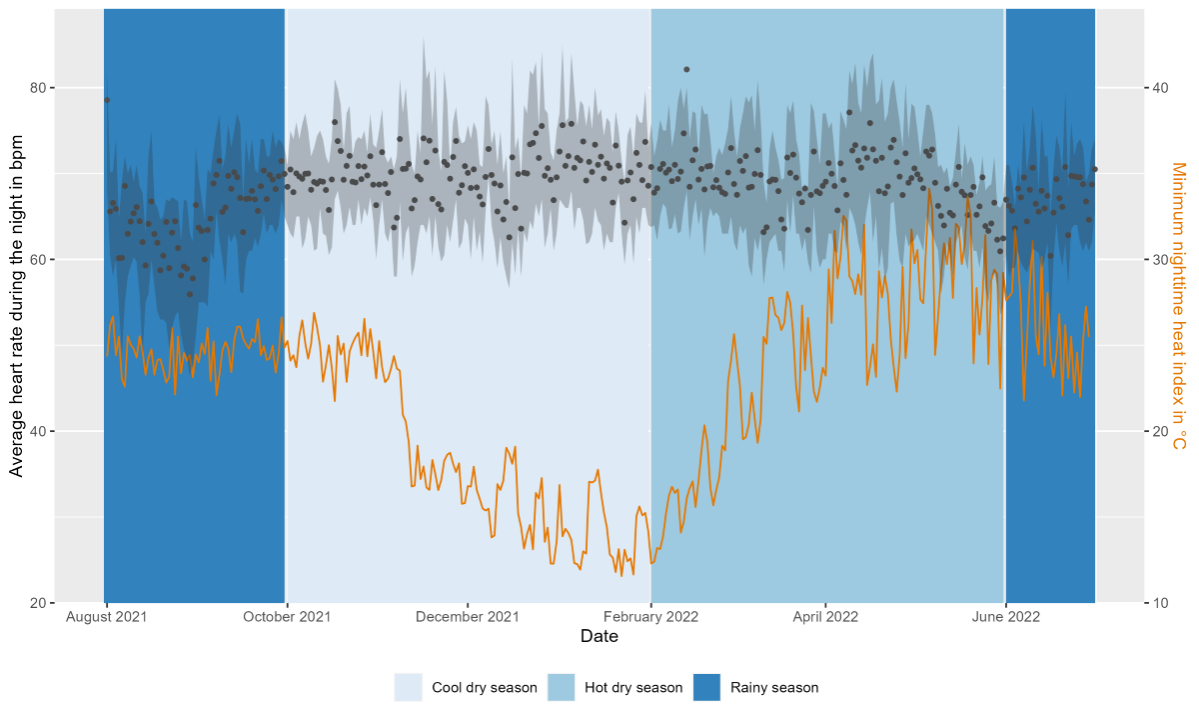
Average nighttime heart rate (beats per minute [bpm]; black dots) with the IQR (gray area) and minimum heat index (°C; orange line; average of all 4 weather stations) over the duration of the study. The background color indicates the 3 seasons (cool dry, hot dry, and rainy).

During the night, the average HR and HI decreased in parallel until the average HR increased again between 5 AM and 6 AM (Figure 9). This pattern was observed in all 3 seasons, although average HR values were lower during the rainy season, whereas HI values were similar between the hot, dry, and rainy seasons (Figure 10).

##### Weather Exposure and HR Measurements

Using a mixed-effects model, we explored the relationship between minimum nighttime HI values (HI_min_) and average nighttime HR. Average nighttime HR showed significant variance in intercepts across participants (*χ^2^*_1_=1030.8, *P*<.001). As HI_min_ and nighttime HR did not show a linear relationship in the residual plots, we added a quadratic term for HI_min_ (HI_min_^2^) to improve the model fit. After adding HI_min_, HI_min_^2^, and total daily precipitation as predictors, we found that neither HI_min_ (*t*_2998_=–0.31; *P*=.75) nor HI_min_^2^ (*t*_2998_=0.46; *P*=.65) as fixed effects were significant. However, the main effect of daily precipitation was significant (*t*_2985_=–1.95; *P*=.009), and we found that, for every 1-mm increase in total daily rainfall, the average nighttime HR decreased by 0.04 bpm (95% CI −0.08 to −0.00). The final model was adjusted for sex, age group, BMI group, and month. Weekends failed to reach statistical significance (*P*<.05) as a covariate. We cross-validated the model using the LOSO approach and calculated *R*^2^ values of up to 0.65, with an average of 0.14. In addition, we created a model using the minimum nighttime temperature in °C as a predictor instead of HI_min_. When comparing both models, we found no better model fit for the minimum nighttime temperature model.

##### Different Heat Sensitivity by Age, BMI, Sex, and Month

We sequentially added an interaction term for age group, BMI group, sex, or month and HI_min_. Interactions for sex, BMI group, and month did not improve the model fit. HR in relation to heat exposure did significantly differ between age groups. As there was only HR data from one young adult (aged <25 years), we only compared middle-aged and older adults. No significant association between HI_min_ and nighttime HR was found either for middle-aged adults (*t*_2642_=–1.41; *P*=.16) or for older adults (*t*_147_=−0.34; *P*=.73).

##### Weather Extremes and HR

To further compare nighttime HR between extreme and nonextreme weather exposures, we created a linear mixed model with the binary predicting factors heat stress night (HI_min_ ≥25 °C) or heavy rainfall (total daily rainfall of ≥20 mm). The models were adjusted for age group, sex, BMI group, and month. Heat stress nights did not have a significant effect on nighttime HR (*t*_2993_=–0.69; *P*=.49). On nights with heavy rainfall, nighttime HR was estimated to be lower by 2 bpm (95% CI −3.81 to −0.21; *t*_2987_=–2.19; *P*=.03) compared with nights with precipitation of <20 mm. The full results of all 3 models can be found in Multimedia Appendix 3.

### Subjective Heat Perception and Adaptation

In response to our questionnaire (with a total response rate of 137/143, 95.8%), almost half (67/137, 48.9%) of the participants reported that heat had an impact on their daily lives. Of the study participants who reported an impact, most reported an impact primarily at night (55/67, 82%), in the afternoon (33/67, 49%), at noon (18/67, 27%), and during outdoor work (15/67, 22%) in the form of poorer sleep (44/67, 66%), sweating (44/67, 66%), exhaustion (27/67, 40%), and fatigue (14/67, 21%).

Most participants (89/137, 65%) stated that they worked outdoors, and some (44/137, 32.1%) worked both indoors and outdoors, whereas only a few (4/137, 2.9%) worked indoors only. When asked about their adaptative measures against heat when they were indoors, the participants said that they left windows or doors open for ventilation (93/137, 67.9%), slept in a cooler place or outdoors (42/137, 30.7%), drank more water (35/137, 25.5%), rested (29/137, 21.2%), did the most strenuous work during cooler times (15/137, 10.9%), or did not take any adaptive measures (23/137, 16.8%). When outdoors, they mostly rested in the shade (79/137, 57.7%), drank more water (77/137, 56.2%), wore loose clothing (37/137, 27%), rested (24/137, 17.5%), used a hat or sun protection (21/137, 15.3%), went inside (19/137, 13.9%), and did the most strenuous work during cooler times (15/137, 10.9%) to protect themselves against the heat. When asked if they were affected by other weather extremes, 21.9% (30/137) of the participants said yes, especially by heavy rains causing flooding of houses, yards, and fields (13/30, 43%); high humidity in the house (8/30, 27%); and destruction of houses (3/30, 10%). A total of 17% (5/30) of the participants mentioned that they were negatively affected by cold temperatures as well.

## Discussion

### Summary of Findings

To investigate the impact of weather on 143 individuals in the rural communities of the Nouna HDSS in Burkina Faso, we used consumer-grade wearables to collect data on their daily activity, sleep, and HR over the course of 11 months. We used weather indexes based on rainfall, temperature, HI, and WBGT estimates to quantify the weather extremes of heavy rainfall, nights with heat stress, and hot days. In addition, we conducted a questionnaire with study participants regarding their perceptions of weather exposure, as well as an activity diary in which study participants were asked to provide retrospective information on their daily activities. We found that sleep duration decreased with higher heat exposure, which corresponds with the participants’ questionnaire responses indicating that they were most affected by the heat at night. In contrast, sleep duration increased with higher precipitation. During the day, most participants (89/137, 65%) worked outdoors doing physical labor in the form of, for example, farming, housework, or fetching water. With increasing WBGT values up to a threshold of approximately 30 °C, daily activity (steps) increased. Increased WBGT above this threshold was associated with decreased daily activity. However, this effect varied by month, with a decrease in daily activity (steps) per degree increase in WBGT during the rainiest month for a WBGT of 20 °C and an increase in activity for a WBGT of ≥30 °C. In addition, increasing precipitation was correlated with lower daily activity (steps). As daytime HR data were limited, we focused primarily on nighttime HR, for which we found that increasing HI had no significant impact. There was a small but statistically significant decrease in the average nighttime HR as rainfall levels rose.

### Daily Activity

Similar to the findings of Edwards et al [53], daily activity in the form of steps increased with higher heat exposure up to a certain threshold. Our study population’s threshold was at WBGT values of 30 °C during most months, which can be considered harmful to health in terms of heat exposure [42]. In general, people seemed to avoid heat exposure, especially during the hot dry season, by deferring outdoor activities until later in the day when temperatures had cooled down and resting during the hottest hours, as indicated by lower step counts during the hottest hours of the day and as reported by the participants in the self-perceived heat questionnaire. In accordance with Al-Mohannadi et al [11], we found that heat stress had no statistically different effects on people of different ages and sex even though women reported less outdoor work in their activity journals. The per-degree effect of WBGT on daily activity (steps) varied between months, with higher absolute step counts and an increase in daily activity observed at a 30 °C WBGT_max_ in the rainiest month (August) compared with a decrease in the coolest (January) and hottest (April) months. A reason for this may be related to the season. Considering the agricultural calendar for Burkina Faso [54], most activities in the heat seemed to take place during usual times of harvest throughout the rainy season. Given the rural Burkinabé population’s dependence on agriculture, many of whom are subsistence farmers who rely on harvest outcomes for their own nutrition and primary source of income [55], it appears that they cannot afford to suspend agricultural activities during extreme heat. Furthermore, higher precipitation was associated with decreased activity, which confirms previous findings [12,56-58].

### Sleep

We found that sleep duration decreased with increasing heat exposure, similar to studies conducted in other countries, including middle-income countries with tropical climates [6,12,56,59]. However, contrary to what has been observed in several other studies, we did not find that overnight heat exposure led to poorer sleep either in older participants compared with younger participants or in female participants compared with male participants [6,59]. The estimated effect of nighttime heat on sleep duration was lowest during the hottest month (April). This might be an indication of people getting used to high air temperatures. In contrast, heat sensitivity was highest during the rainiest and most humid month (August), which matches the findings that humans are affected more by heat when relative humidity is higher compared with higher air temperature alone [5]. On half of the nights evaluated, sleep duration was insufficient, and overall averages were up to an hour lower than those observed in studies conducted in other countries [60]. Our estimates of sleep duration did not provide insights into changes in sleep physiology. However, it has been found that rapid eye movement sleep decreases with higher ambient temperatures in laboratory settings [61]. Moreover, the wearables did not capture sleep for <3 hours. As such, we could only evaluate nighttime sleep as opposed to shorter naps. As a result, it is unclear whether individuals were able to make up for nighttime sleep deficits by taking naps throughout the day. Increasing precipitation was associated with increasing sleep duration in accordance with the findings of the large-scale study by Minor et al [6].

### HR Findings

In contrast to earlier findings [62,63], average HR values were lower in our study when individuals were exposed to increasing heat; however, this effect was not statistically significant. Furthermore, it is important to consider that we looked at average nighttime HR, which reflects resting HR, and not HR during activities, as in other studies. It must also be noted that our analysis was based on a very small data set owing to the low data completeness for HR. With the large proportion of missing data, our study results should only be considered as hypothesis generating [47]. The lower HR values during nights with higher HI_min_ may indicate heat adaptation of study participants [64]. The nighttime HR was significantly influenced by the amount of rainfall that occurred each day. When the total amount of rainfall was greater, as it often is during severe rains, HR values were slightly lower. These results corroborate the earlier observation that more frequent and heavier rainfall is associated with longer periods of sleep, indicating better rest. As the cardiovascular system is severely affected by life-threatening events such as heatstrokes, it would be essential to conduct additional research on the cardiovascular effects of climate change and extreme weather exposure.

### Weather Exposure

Although the highest temperatures often occurred during the dry months, WBGT and HI readings peaked during the rainy season. During our study, WBGT estimates frequently reached values of >30 °C, which is considered critical even under moderate activity [42]. A total of 8 days of heavy rain were experienced by the participants throughout the rainy season. Participants ranked heavy rainfall and flooding as the second most impactful weather event. The number of floods has been increasing over the past few decades in West Africa [22,65] and is a contributing factor to food insecurity and economic loss for subsistence farmers [28]. The rural study population in Nouna, Burkina Faso, is highly exposed to weather extremes in the form of heat and heavy rains, which will likely increase in the future because of climate change [66,67].

Although WBGT was originally introduced as a measure for heat stress for outdoor work in direct sunlight, our model comparison showed that WBGT predicted daily activity better than temperature alone. A reason may be that humidity, a component of WBGT and HI, is an important indicator of a person’s heat stress [5]. We calculated WBGT based on weather station measurements, but it should be noted that Lemke and Kjellstrom [68] have found no statistically significant difference between calculated and measured WBGT values. Nevertheless, the assessment of heat exposure using WBGT has its limitations as it, for example, does not include adjustment for clothing [69]. Furthermore, we calculated WBGT based on the equation by Carter et al [39], which was based on cooler climatic conditions than those in our study setting. Therefore, WBGT values should be considered estimates.

### Self-Perceived Burden of Heat Exposure

Half (67/137, 48.9%) of the participants reported being negatively affected by heat, and some (30/137, 21.9%) also mentioned heavy rains and flooding as impairing factors in their daily lives. They experienced most disturbances at night, during the hottest times of the day, and during outdoor work. This was also reflected in our objective measurements. The participants already reported many adaptive measures when outdoors and indoors that are also recommended by the World Health Organization [70]. This included resting, seeking shade, working during cooler times of the day, sleeping outdoors or in cooler places, and leaving doors and windows open for ventilation. This adaptive behavior could be seen in the results of their activity journals as well, where they reported most of the outdoor and strenuous work in the morning and more resting during midday and the afternoon, when heat exposure was highest. There is an immediate need for more adaptive and preventive measures for this low-resource population as the future poses even more severe climate conditions.

### Limitations

One of the major limitations of our study was incomplete data. Data completeness was low for both accelerometry (daily activity and sleep) and photoplethysmography (HR) data. This could be due to multiple reasons. Environmental factors had a significant impact on reaching the study participants in their homes and synchronizing the wearable data. During the rainy season, for instance, certain villages were inaccessible because of flooding, making many dirt roads impassable for fieldworkers. In addition, the political environment in Burkina Faso hindered the collection of data because of security concerns in some villages (threats of terrorism) and the shutdown of mobile internet in November 2021 and during the coup d’état in January 2022. The limitation of internet connection in most of the study area presented a barrier to the synchronization of participant data at their homes. Another main limitation was damaged wearables. Over the course of the study, >40 devices malfunctioned, of which 20 could be replaced. The most common causes of wearables breaking were water damage (although the devices were waterproof) and the impact of force (eg, during fieldwork). Second, the lack of information regarding the validity of the Withings Pulse HR is a major limitation. The validity of these devices has not been verified in a study setting. Therefore, we can only consider the values as estimates. As Withings only released the processed accelerometry data and not the raw accelerometry data, we lacked clarity regarding the calculations that underpinned these data points for sleep and daily activity. Furthermore, we deemed the pulse rate measured by the photoplethysmography sensor equivalent to HR. The technical aspects of the wearables could have also caused low data completeness and inaccuracy, especially for HR measurements. In previous studies, various noise sources have been identified that impaired the photoplethysmography signals and caused inaccurate measurements [71]. This includes individual, external, and physiological factors such as obesity, skin tone, body site, and motion artifacts. Owing to its relatively narrow photoplethysmography sensor compared with other consumer-grade wearables, the Withings Pulse HR may not have accurately measured participants’ HRs while they were in motion or sweating heavily. Furthermore, it has been suggested that melanin disrupts the functionality of photoplethysmography, resulting in poorer performance on darker skin tones, which could explain the low data completeness in our study population [72,73]. To match weather and wearable data, we used weather data from the nearest weather station to each participant’s house, not considering when participants were not at home, which might have caused inaccuracy. Regarding the representativeness of our study cohort, we had one major limitation: most of our study population was middle-aged even though most Burkinabé are aged <25 years [74]. It would be important to gain more insights into the sensitivity of children and young adults to heat and heavy rains. Finally, the linear mixed-effects models that we used to explore the relationship between extreme weather exposure and health parameters had some limitations. We tried to address possible overfitting through hierarchical model building and cross-validation. However, the models showed quite low average *R*^2^ values when cross-validated using the LOSO approach. We still deemed our models’ performance sufficient for this exploratory analysis as *R*^2^ values for some participants’ data as test data were much higher, especially for those participants with good data completeness, which leads us back to our first major limitation.

### Conclusions

On the basis of our findings, the rural population in Burkina Faso is exposed to many days of extreme weather in the form of heavy rains, nights with heat stress, and hot days. Heat especially seemed to be associated with shortened sleep duration, which was also confirmed by the subjective perceptions of study participants, who reported that heat had the greatest impact on their daily lives at night. Heat-related sleep disruption is a major issue for the general public’s health. During the hottest month (April), daily activity (steps) decreased with increased heat exposure at 30 °C WBGT. Total daily activity (steps) was highest during the rainy season, typically June to September, which had the highest number of heat stress days, and the number of steps even increased as WBGT rose above 30 °C. Most people in rural Burkina Faso are subsistence farmers who depend on their harvests for food and income. This means that most of the agricultural work, especially during the rainy season when most crops are harvested, may coincide with the most extreme weather exposure. Other essential activities such as obtaining water and caring for livestock also potentially expose Burkinabés to weather conditions that may be detrimental to their health. Heavy rainfall was associated with a small increase in sleep duration, slightly decreased average nighttime HR, and decreased daily activity (steps). Participants in the study also recognized the agricultural and economic impacts of heavy rainfall as threats to their daily lives, which should be explored in future research. Study participants reported adverse health impacts of weather and adaptive measures, such as avoiding extreme heat and delaying physical activities until cooler times. This is the first long-term study that, to our knowledge, evaluates the impacts of weather exposure on a rural population in Burkina Faso using objective measures from consumer-grade wearables to better comprehend everyday exposure. On the basis of these findings, new adaptive measures could be implemented in rural, low-resource communities that are highly exposed to climate change to protect people from heat, especially during the night and outdoor work. This could be, for example, in the form of housing cooling methods or personal protective gear.

## Appendix

Multimedia Appendix 1 STROBE (Strengthening the Reporting of Observational Studies in Epidemiology) statement: guidelines for reporting observational studies.

Multimedia Appendix 2 Activity journal and heat questionnaire.

Multimedia Appendix 3 Full results of the linear mixed-effects models.

## Abbreviations

bpm: beats per minute
HDSS: health and demographic surveillance system
HI: heat index
HR: heart rate
LOSO: leave-one-subject-out
STROBE: Strengthening the Reporting of Observational Studies in Epidemiology
WBGT: wet-bulb globe temperature

## Data Availability

The data sets generated during the study and the corresponding R scripts can be made available from the corresponding author in an anonymized form upon individual request.
