# Supplementary material for: Assessing the Effect of Extreme Weather on Population Health Using Consumer-Grade Wearables in Rural Burkina Faso: Observational Panel Study
Source: JMIR Mhealth Uhealth. 2023 Nov 8;11:e46980. doi: 10.2196/46980 (PMC10666008; doi:10.2196/46980)
Supplement: Multimedia Appendix 2 [file mhealth_v11i1e46980_app2.pdf]

Generated by vboudo, Jan 31, 2022 17:27
Questionnaire created by cp1, Jun 28, 2021 14:53
Last modified by vboudo, Jan 31, 2022 17:27

Shared with:
vboudo last edited 1/31/2022 5:27:45 PM

Sections: 4, Sub-sections: 0, Questions: 68.
Questions with enabling conditions: 14
Questions with validation conditions:0
Rosters: 2
Variables: 0

CP1- P5 Étude complète Burkina Faso

SURVEY IDENTIFICATION INFORMATION
QUESTIONNAIRE DESCRIPTION

DÉTAILS DES PARTICIPANTS À L'ÉTUDE
No sub-sections, No rosters, Questions: 3, Static texts: 1.

DISTRIBUTION/RETOUR DE CAPTEUR
No sub-sections, Rosters: 2, Questions: 28.

QUESTIONNAIRE D'ACCEPTATION
No sub-sections, No rosters, Questions: 29, Static texts: 1.

JOURNAL D'ACTIVITÉ
No sub-sections, No rosters, Questions: 8.

APPENDIX A — CATEGORIES

LEGEND

SURVEY IDENTIFICATION INFORMATION QUESTIONNAIRE DESCRIPTION

Basic information

Title	CP1- P5 Étude complète Burkina Faso

Survey data information

Study type	Integrated Survey
Kind of data	Aggregate data [agg]

Survey information

Country	Burkina Faso
Year	2020
Languages	French
Funding	DFG

JOURNAL D'ACTIVITÉ

Quelle activité avez-vous fait après vous être levé ce matin? (choix multiple)	MULTI-SELECT: ORDERED `after_gettingup`<br>01 ☐ travaillant à la ferme<br>02 ☐ cuisine<br>03 ☐ vendre des produits sur le marché<br>04 ☐ élevage d'animaux<br>05 ☐ prendre soin des enfants<br>06 ☐ prendre soin de son mari / femme<br>07 ☐ prendre soin des membres de la famille<br>08 ☐ va à l'école<br>09 ☐ chercher de l'eau dans le puits<br>10 ☐ récolte<br>11 ☐ travailler sur le terrain<br>12 ☐ je me reposais<br>13 ☐ regarder la télévision<br>14 ☐ aller au bar<br>15 ☐ aller au restaurant<br>16 ☐ faire du sport<br><br>And 5 other symbols [3]
Quelle activité avez-vous pratiquéz le matin? (choix multiple)	MULTI-SELECT `morning_activity`<br>01 ☐ travaillant à la ferme<br>02 ☐ cuisine<br>03 ☐ vendre des produits sur le marché<br>04 ☐ élevage d'animaux<br>05 ☐ prendre soin des enfants<br>06 ☐ prendre soin de son mari / femme<br>07 ☐ prendre soin des membres de la famille<br>08 ☐ va à l'école<br>09 ☐ chercher de l'eau dans le puits<br>10 ☐ récolte<br>11 ☐ travailler sur le terrain<br>12 ☐ je me reposais<br>13 ☐ regarder la télévision<br>14 ☐ aller au bar<br>15 ☐ aller au restaurant<br>16 ☐ faire du sport<br><br>And 5 other symbols [3]

Quelle activité as-tu pratiquée le midi? (choix multiple)	MULTI-SELECT<br>01 ☐ travaillant à la ferme<br>02 ☐ cuisine<br>03 ☐ vendre des produits sur le marché<br>04 ☐ élevage d'animaux<br>05 ☐ prendre soin des enfants<br>06 ☐ prendre soin de son mari / femme<br>07 ☐ prendre soin des membres de la famille<br>08 ☐ va à l'école<br>09 ☐ chercher de l'eau dans le puits<br>10 ☐ récolte<br>11 ☐ travailler sur le terrain<br>12 ☐ je me reposais<br>13 ☐ regarder la télévision<br>14 ☐ aller au bar<br>15 ☐ aller au restaurant<br>16 ☐ faire du sport<br><br>And 5 other symbols [3]	noon_activity
Quelle activité avez-vous faite l'après-midi? (choix multiple)	MULTI-SELECT<br>01 ☐ travaillant à la ferme<br>02 ☐ cuisine<br>03 ☐ vendre des produits sur le marché<br>04 ☐ élevage d'animaux<br>05 ☐ prendre soin des enfants<br>06 ☐ prendre soin de son mari / femme<br>07 ☐ prendre soin des membres de la famille<br>08 ☐ va à l'école<br>09 ☐ chercher de l'eau dans le puits<br>10 ☐ récolte<br>11 ☐ travailler sur le terrain<br>12 ☐ je me reposais<br>13 ☐ regarder la télévision<br>14 ☐ aller au bar<br>15 ☐ aller au restaurant<br>16 ☐ faire du sport<br><br>And 5 other symbols [3]	afternoon_activity

Quelle activité as-tu pratiquée le soir? (choix multiple)	MULTI-SELECT evening_activity<br>01 ☐ travaillant à la ferme<br>02 ☐ cuisine<br>03 ☐ vendre des produits sur le marché<br>04 ☐ élevage d'animaux<br>05 ☐ prendre soin des enfants<br>06 ☐ prendre soin de son mari / femme<br>07 ☐ prendre soin des membres de la famille<br>08 ☐ va à l'école<br>09 ☐ chercher de l'eau dans le puits<br>10 ☐ récolte<br>11 ☐ travailler sur le terrain<br>12 ☐ je me reposais<br>13 ☐ regarder la télévision<br>14 ☐ aller au bar<br>15 ☐ aller au restaurant<br>16 ☐ faire du sport<br><br>And 5 other symbols [3]
Quelle activité as-tu pratiquée la nuit? (choix multiple)	MULTI-SELECT night_activity<br>01 ☐ travaillant à la ferme<br>02 ☐ cuisine<br>03 ☐ vendre des produits sur le marché<br>04 ☐ élevage d'animaux<br>05 ☐ prendre soin des enfants<br>06 ☐ prendre soin de son mari / femme<br>07 ☐ prendre soin des membres de la famille<br>08 ☐ va à l'école<br>09 ☐ chercher de l'eau dans le puits<br>10 ☐ récolte<br>11 ☐ travailler sur le terrain<br>12 ☐ je me reposais<br>13 ☐ regarder la télévision<br>14 ☐ aller au bar<br>15 ☐ aller au restaurant<br>16 ☐ faire du sport<br><br>And 5 other symbols [3]
Veuillez ajouter l'activité (ainsi que l'heure de la journée) et décrire, si possible	TEXT comment_activity<br>..
Coordonnées GPS du Menage	GPS HH_GPS<br>- - - - - - - - - - - -<br>N<br>- - - - - - - - - - - -<br>W<br>- - - - - - - - - - - -<br>A

APPENDIX A — CATEGORIES

[1] Categories_LikertScale
Categories: 1:Tout à fait d'accord, 2:D'accord, 3:Ni en désaccord ni d'accord, 4:Pas d'accord, 5:Pas du tout d'accord

[2] Categories_Sensors
Categories: 1:Withings Pulse HR (noir poignet), 2:Tucky thermomètre (blanc tache), 3:Aucune

[3] Categories_Activity_Diary
Categories: 1:travaillant à la ferme, 2:cuisine, 3:vendre des produits sur le marché, 4:élevage d'animaux, 5:prendre soin des enfants, 6:prendre soin de son mari / femme, 7:prendre soin des membres de la famille, 8:va à l'école, 9:chercher de l'eau dans le puits, 10:récolte, 11:travailler sur le terrain, 12:je me reposais, 13:regarder la télévision, 14:aller au bar, 15:aller au restaurant, 16:faire du sport, 17:conduire un véhicule, 18:travaux ménagers, 19:j'ai fait du shopping, 20:nettoyer, 21:travailler assis

[4] nom_village: Nom du village du participant
Categories: 1:Barakuy, 2:Toni, 3:Biron Bobo, 4:Biron Marka, 5:Boron, 6:Bouni/Boune, 7:Bourasso, 8:Cisse, 9:Dankoumana, 10:Dembelela, 11:Denissa, 12:Denissa Mossi, 13:Diamasso, 14:Dionkongo, 15:Dina, 16:Dokoura, 17:Goni, 18:Kamadena, 19:Kemena, 20:Kodougou, 21:Koro, 22:Labarani, 23:Lei, 24:Lekuy, 25:Lemini, 26:Nokuy, 27:Ouette, 28:Pa, 29:Sampopo, 30:Seriba, 31:Sien, 32:Sikoro, 33:Sobon, 34:Solimana, 35:Sirakoro/Sirakorosso, 36:Tebere, 37:Tonsere, 38:Zanakuy, 39:Tissi, 40:Dara, 41:Bankoumani1, 43:Babekolon, 44:Bagala, 45:Biron badala, 46:Bissau, 47:Bokuy, 48:Damandigui, 49:Hinkuy, 50:Kamiankoro, 51:Kansara, 52:Kerena, 53:Konkuini, 54:Koredougou, 55:Moinsi, 56:Mourdie, 57:Sere, 58:Soin, 59:Tonkoroni, 60:BABAKUY, 62:BANGASSI-KORO, 63:BANGASSI_KOUROU, 64:BARANI, 65:NOUNA, 66:BOGO3, 67:BOULEMPORO, 68:BOULE, 69:DIAMAHOUN, 70:DIENWELY, 71:DJALLO, 72:DOURE, 73:GNIMANOU, 74:ILLA, 75:KAMANDADOUGOU, 76:KAREKUY3, 77:KESSEKUY, 78:KINSERE, 79:KOLONKAN GOURE BA, 80:KOLONKAN GOURE DIALLO, 81:KONKORO, 82:KORONI, 83:KOUBE, 84:KOULEROU, 85:MANEKUY, 86:MANTAMOU, 87:MEDOUGOU, 88:NABASSO, 89:NIAKO, 91:OUEMBOYE, 92:OUERESSE, 93:PAMPAKUY, 94:SEKUY, 95:SEKUY-IRA, 96:SOKOURA3, 97:SOUDOGO, 98:TIRA, 99:TOROKOTO, 100:WARIBERE, 101:YALANKORO, 102:BANAKORO, 103:BOGO4, 104:BOMBOROKUY, 105:BOREKUY, 106:DANEKUY, 107:GOMBELE, 108:KOMONKUY, 109:MARIASSO, 110:NIANKOUINI, 111:SADIGAN, 112:SAKO, 113:SOUANKUY, 114:TIRAKUY, 115:YABANA, 116:YALLO, 117:BA, 118:BANANA, 119:BANKOUMANA, 120:BARA, 121:BERKOUE, 122:BIDA, 123:BOKORO, 124:BONOUA, 125:BOUAKUY, 126:BOURIO, 127:DIEKUINI, 128:DIENA, 129:DIEKAN, 130:DJIBASSO, 131:DONKORO, 132:FONI-BORONKIN, 133:GNIMINI, 134:IRA, 135:KANSARA, 136:KIENEKUY, 137:KIRA, 138:KIE, 139:KOLONKAN, 140:KOLONKANI-SIRAKORO, 141:KOLONZO5, 142:KOMBORI5, 143:MANDARA, 144:MAOULENA, 145:MASSAKUY, 146:MOUNA, 147:NAIRENA, 148:OUAROKUY, 149:OURA, 150:OUROUKO, 151:PARAKUY, 152:PARANZO, 153:PIA N 1, 154:SABA, 155:SADIGNAKONO, 156:SAKUY, 157:SAMEKUY, 158:SARAKORO, 159:SENOULO, 160:SIEDOUGOU, 161:SOUMOUKUY, 162:SOUNE, 163:SOYE, 164:TIEME, 165:VORO, 166:AYOUBAKOLON, 167:BONIKUY, 168:DAR-ES-SALAM, 169:DASSI, 170:DOKUY, 171:DOUBALE, 172:GASSINGO, 173:ILABEKOLON, 174:KANADOUGOU, 175:KARASSO, 176:KEMENSO, 177:KENEKUY, 178:KOLONIDARA, 179:KOLONKOURA6, 180:MAKUY, 181:NEREKO, 182:SOKOURA6, 183:SOUM, 184:SOUMAKORO, 185:TOMIKORONI, 186:BAMPERLA, 187:BANGASSI-BOBO, 188:BANGASSI-ILLA, 189:BANGASSI-MAMOUDOUD, 190:BANKUY, 191:BASSAM, 192:BOANEKUY, 193:BOKUY7, 194:BOUKUY, 195:DAKUY, 196:DOUMBALA, 197:HENLEKUY, 198:KAREKUY7, 199:KIMBA, 200:KINI-KINI, 201:KOA, 202:KODARA, 203:KOLONZO7, 204:KONKUY-BOHO, 205:KONKUY-KORO, 206:KOURKUY, 207:LANFIERA, 208:MONTIONKUY, 209:MOUNAKORO, 210:NIAN, 211:POROKUY, 212:SAINT-CAMILLE, 213:SAINT-MARTIN, 214:SAINT-PAUL, 215:SAWOROKUY, 216:SIMBORA, 217:TENI, 218:TENI-PEULH, 219:TIOURKUY, 220:WANZAN, 221:ZEKUY, 222:ABAYE, 223:AOUREMA8, 224:BA-PEULH, 225:DAGA, 226:GANI, 227:KOLONKANI-BA, 228:KOMBORI8, 229:KONNA, 230:LONANI, 231:MAGADIAN, 232:OUORI, 233:SANAKADOUGOU, 234:SASSAMBARI, 235:SIEKORO, 236:SIEWALI, 237:SIGUIDE, 238:YARAN, 239:BANKOUMANI, 240:BOKUY9, 241:DINA, 242:KIKO, 243:KOLOKAN, 244:MADOUBA, 245:PIA N 2, 246:PORO, 247:TOUBA, 248:YOUROUNA, 249:AOUREMA1, 250:BARE, 251:DANTIERA, 252:DEMBO, 253:DIGANI, 254:DIONDOUGOU, 255:FARAKUY, 256:KAKI, 257:KALFADOUGOU, 258:KAREKUY1, 260:KOMBARA, 261:KONANKOIRA, 262:KONONIBA, 263:MANI, 264:PATIARAKUY, 265:SAINT-JEAN, 266:SAINT-LOUIS, 267:SIMBADOUGOU, 268:SOA, 269:SOKORO, 270:TENOU, 271:THIA, 272:TOMBODOUGOU, 273:ZOUN, 275:BANTOMBO, 276:BOTTE, 277:KALLE, 278:KOURY, 279:LANFIERA-KOURA, 280:SIELA, 281:SONO, 282:SORO, 283:ZAMPANA, 285:sontorokuy, 286:yevedougou, 288:Homokuy, 289:Daborokuy, 290:Warkuy, 291:Koncoba, 292:Sayokuy, 293:Poe, 295:Werimbere, 296:Noumbere

LEGEND

Legend and structure of information in this file

Name of section

Enabling condition for this section

Question title

Type of question, scope

Answer options

Variable name

SECTION 5: OTHER INCOME SOURCES

E s4_other_sources_which.Contains(98)

Duis aute irure dolor in reprehenderit in voluptate velit esse cillum dolore eu fugiat nulla pariatur?

I This refers to family relations
E s3_time_other > 0
V1 s4_rel_leaders_which.Contains(98)
M1 Can not be itself
V2 (s3_time_other_breeding_advice <= (50 - s3_time_art_insem_advice)) ||s3_time_other_breeding_advice == 0
M2 This person is not in the list
F optioncode != s5_ignored_option_code

MULTI-SELECT
SCOPE: PREFILLED

s4_rel_leaders_other

01 ☐ Community animal health workers
02 ☐ Private
03 ☐ Government
04 ☐ Livestock keepers association
05 ☐ NGO

And 5 other [13]

Additional information:
"I" — Question instruction
"E" — Enabling condition
"V1" — Validation condition №1
"M1" — Message for validation №1
"F" — Filter in Categorical questions

Link to full set in appendix

Breadcrumbs

Type or roster

Roster Title

CHAPTER 3 IDENTIFICATION /
Roster: LEADER RELATION DETAILS
generated by fixed list:

01 Ward Livestock Officer
02 Village Livestock Officer
99 Other (specify)

List items

Generated by MaraK, Sep 08, 2022 11:09
Questionnaire created by vboudo, Jul 28, 2022 12:26
Last modified by vboudo, Aug 02, 2022 12:42

Shared with:
RUguillaume (never edited)
sandrabarteit (never edited)
MaraK (never edited)

Sections: 2, Sub-sections: 0, Questions: 15.
Questions with enabling conditions: 7
Questions with validation conditions:0
Rosters: 0
Variables: 0

Survey Solutions

Questionnaire sur la chaleur CP1-P5

SURVEY IDENTIFICATION INFORMATION
QUESTIONNAIRE DESCRIPTION

DÉTAILS DES PARTICIPANTS À L'ÉTUDE
No sub-sections, No rosters, Questions: 2.

INFORMATION SUR LA CHALEUR
No sub-sections, No rosters, Questions: 13.

APPENDIX A — CATEGORIES

LEGEND

SURVEY IDENTIFICATION INFORMATION QUESTIONNAIRE DESCRIPTION

Basic information

Title	Questionnaire sur la chaleur CP1-P5

Survey data information

Study type	Integrated Survey
Kind of data	Aggregate data [agg]

Survey information

Country	Burkina Faso
Year	2020
Languages	French
Funding	DFG

DÉTAILS DES PARTICIPANTS À L'ÉTUDE

study_part_details

Fournir l'ID du participant à l'étude<br><br>I Veuillez Saisir l'identifiant du participant	TEXT study_part_id<br><br>..
Nom du village du participant	SINGLE-SELECT: COMBO BOX nom_village<br>02 O Toni<br>05 O Boron<br>08 O Cissé<br>09 O Dankoumana<br>10 O Dembeléla<br>11 O Denissa<br>12 O Denissa Mossi<br>14 O Dionkongo<br>15 O Dina<br>16 O Dokoura<br>17 O Goni<br>18 O Kamadena<br>19 O Kèmena<br>21 O Koro<br>23 O Leï<br>27 O Ouetté<br><br>And 43 other symbols [4]

INFORMATION SUR LA CHALEUR

info_ch

Pour certaines personnes, leur corps se sent stressé pendant la chaleur et ils ont l'impression que cela les affecte. Considérez-vous la chaleur comme un problème pour votre santé et votre corps dans votre vie courante?	SINGLE-SELECT ch1<br>01 ○ Oui<br>02 ○ Non
Si oui, dans quelle situation cela vous impacte-t-il le plus?<br>E ch1==1	MULTI-SELECT ch2<br>01 ☐ Pendant la nuit<br>02 ☐ Du matin<br>03 ☐ A midi<br>04 ☐ A l'après-midi<br>05 ☐ Dans la soirée<br>06 ☐ Pendant le travail à l'extérieur<br>07 ☐ Pendant le travail à l'intérieur de la maison<br>08 ☐ En allant chercher de l'eau<br>09 ☐ Pendant un travail épuisant/exigeant<br>10 ☐ Autre
Autre a preciser<br>E ch2.Contains(10)	TEXT ch2_autre<br>..
Comment la chaleur vous affecte-t-elle?<br>E ch1==1	MULTI-SELECT ch3<br>01 ☐ épuisement<br>02 ☐ transpiration<br>03 ☐ mauvais sommeil<br>04 ☐ fatigue<br>05 ☐ Autre
Autre a preciser<br>E ch3.Contains(5)	TEXT ch3_autre<br>..

Que faites-vous pour vous protéger de la chaleur lorsque vous êtes à l'intérieur/à la maison?	MULTI-SELECT ch4<br>01 ☐ Rien<br>02 ☐ Boire plus d'eau<br>03 ☐ Se reposer<br>04 ☐ Effectuer les travaux les plus pénibles pendant les heures les plus fraîches de la journée (le matin, le soir, etc.) ou par une journée plus fraîche<br>05 ☐ Laisser la porte/fenêtre ouverte pour la ventilation<br>06 ☐ Dormir dans un endroit plus frais/à l'extérieur<br>07 ☐ Aller dans un lieu public climatisé<br>08 ☐ Se baigner fréquemment<br>09 ☐ Mouiller les vêtements<br>10 ☐ Planter des arbres/plantes près de la maison pour plus de fraîcheur/ombre<br>11 ☐ Utilisez des stores/rideaux pour garder la maison fraîche<br>12 ☐ Rester à l'intérieur<br>13 ☐ Utilisez un ventilateur<br>14 ☐ Autre
Autre a preciser<br>E `ch4.Contains(14)`	TEXT ch4_autre<br>..
Que faites-vous pour vous protéger de la chaleur lorsque vous êtes à l'extérieur	MULTI-SELECT ch5<br>01 ☐ Rien<br>02 ☐ Rester à l'ombre<br>03 ☐ Boire plus d'eau<br>04 ☐ Se reposer<br>05 ☐ Effectuer les travaux les plus pénibles pendant les heures les plus fraîches de la journée (le matin, le soir, etc.) ou par une journée plus fraîche<br>06 ☐ Utiliser un chapeau/une protection solaire<br>07 ☐ Aller à l'intérieur<br>08 ☐ Aller dans un lieu public climatisé<br>09 ☐ Se baigner fréquemment<br>10 ☐ Mouiller les vêtements<br>11 ☐ Portez des vêtements légers<br>12 ☐ Utilisez un ventilateur<br>13 ☐ Autre
Autre a preciser<br>E `ch5.Contains(13)`	TEXT ch5_autre<br>..

Travaillez-vous principalement à l'intérieur ou à l'extérieur?	SINGLE-SELECT ch6<br>01 O à l'intérieur<br>02 O à l'extérieur<br>03 O Les deux (intérieur et extérieur)
Y a-t-il d'autres conditions météorologiques extrêmes qui vous affectent négativement ?	SINGLE-SELECT ch7<br>01 O Oui<br>02 O Non
si oui, comment? (par exemple, inondation lors de fortes pluies) Notez la météo et l'effet sur le participant s'il vous plaît.<br>E ch7==1	TEXT ch8<br>..
Coordonnées GPS du menage du participant	GPS ch7_gps<br>- - - - - - - - - - - - - - - - - - - -<br>N<br>- - - - - - - - - - - - - - - - - - - -<br>W<br>- - - - - - - - - - - - - - - - - - - -<br>A

APPENDIX A — CATEGORIES

[1] Categories_LikertScale
Categories: 1:Tout à fait d'accord, 2:D'accord, 3:Ni en désaccord ni d'accord, 4:Pas d'accord, 5:Pas du tout d'accord

[2] Categories_Sensors
Categories: 1:Withings Pulse HR (noir poignet), 2:Tucky thermomètre (blanc tache), 3:Aucune

[3] Categories_Activity_Diary
Categories: 1:travaillant à la ferme, 2:cuisine, 3:vendre des produits sur le marché, 4:élevage d'animaux, 5:prendre soin des enfants, 6:prendre soin de son mari / femme, 7:prendre soin des membres de la famille, 8:va à l'école, 9:chercher de l'eau dans le puits, 10:récolte, 11:travailler sur le terrain, 12:je me reposais, 13:regarder la télévision, 14:aller au bar, 15:aller au restaurant, 16:faire du sport, 17:conduire un véhicule, 18:travaux ménagers, 19:j'ai fait du shopping, 20:nettoyer, 21:travailler assis

[4] nom_village: Nom du village du participant
Categories: 2:Toni, 5:Boron, 8:Cissé, 9:Dankoumana, 10:Dembeléla, 11:Denissa, 12:Denissa Mossi, 14:Dionkongo, 15:Dina, 16:Dokoura, 17:Goni, 18:Kamadena, 19:Kèmena, 21:Koro, 23:Leï, 27:Ouetté, 28:Pa, 29:Sampopo, 30:Seriba, 31:Sien, 33:Sobon, 34:Solimana, 36:Tebéré, 37:Tonséré, 39:Tissi, 40:Dara, 41:Bankoumani, 42:Nouna, 43:Babekolon, 44:Bagala, 45:Biron badala, 46:Bissau, 47:Bokuy, 48:Damandigui, 49:Hinkuy, 51:Kansara, 52:Kéréna, 53:Konkuini, 54:Korédougou, 55:Moinsi, 56:Mourdié, 57:Sèrè, 58:Soin, 59:Tonkoroni, 1:Barakuy, 3:Biron-Bobo, 4:Biron-Marka, 6:Bouni/Bouné, 7:Bourasso, 13:Diamasso, 20:Kodougou, 22:Labarani, 24:Lekuy, 25:Lemini, 26:Nokuy, 32:Sikoro, 35:Sirakoro/Sirakorosso, 38:Zanakuy, 50:Kamiankoro

LEGEND

Legend and structure of information in this file

Name of section

Enabling condition for this section

Question title

Type of question, scope

Answer options

Variable name

SECTION 5: OTHER INCOME SOURCES

E s4_other_sources_which.Contains(98)

Duis aute irure dolor in reprehenderit in voluptate velit esse cillum dolore eu fugiat nulla pariatur?

I This refers to family relations
E s3_time_other > 0
V1 s4_rel_leaders_which.Contains(98)
M1 Can not be itself
V2 (s3_time_other_breeding_advice <= (50 - s3_time_art_insem_advice)) ||s3_time_other_breeding_advice == 0
M2 This person is not in the list
F optioncode != s5_ignored_option_code

MULTI-SELECT
SCOPE: PREFILLED

s4_rel_leaders_other

01 ☐ Community animal health workers
02 ☐ Private
03 ☐ Government
04 ☐ Livestock keepers association
05 ☐ NGO

And 5 other [13]

Additional information:
"I" — Question instruction
"E" — Enabling condition
"V1" — Validation condition №1
"M1" — Message for validation №1
"F" — Filter in Categorical questions

Link to full set in appendix

Breadcrumbs

Type or roster

Roster Title

CHAPTER 3 IDENTIFICATION /
Roster: LEADER RELATION DETAILS
generated by fixed list:

01 Ward Livestock Officer
02 Village Livestock Officer
99 Other (specify)

List items
