# Supplementary material for: Assessing the Effect of Extreme Weather on Population Health Using Consumer-Grade Wearables in Rural Burkina Faso: Observational Panel Study
Source: JMIR Mhealth Uhealth. 2023 Nov 8;11:e46980. doi: 10.2196/46980 (PMC10666008; doi:10.2196/46980)
Supplement: Multimedia Appendix 3 [file mhealth_v11i1e46980_app3.pdf]

Table S1. Results of Model 1 (WBGT or HI and total daily precipitation as predictors), Model 2 (temperature and total daily precipitation as predictors) and Model 3 (binary variable heat stress day/night as predictor) for daily activity (steps), sleep duration and nighttime HR. For the outcome of sleep duration Model 1 had an AIC of 76228, Model 2 an AIC of 76230 and Model 3 an AIC of 76309. For the outcome steps Model 1 had an AIC of 454907, Model 2 had an AIC of 454947 and Model 3 had an AIC of 454941. For the outcome variable nighttime HR Model 1 had an AIC of 21049, Model 2 had an AIC of 21049 and Model 3 had an AIC of 21047.

		Model 1			Model 2			Model 3		
Independent	Predictor	Estimate	SE	p-value	Estimate	SE	p-value	Estimate	SE	p-value
Sleep duration (h)	Intercept	8.11	0.10	<.001	8.24	0.11	<.001	7.51	0.08	<.001
	HI_{min}	-0.04	0.00	<.001						
	Daily Precipitation	0.01	0.00	<.001	0.01	0.00	<.001			
	$Temp_{min}$				-0.05	0.00	<.001			
	Heat stress night (HI_{min}≥25°C)							-0.24	0.03	<.001
	HI_{min}<25°C							Ref	Ref	Ref
	August 2021	-0.41	0.07	<.001	-0.46	0.07	<.001	-0.67	0.05	<.001
	September 2021	-0.72	0.07	<.001	-0.77	0.07	<.001	-1.05	0.05	<.001
	October 2021	-0.56	0.07	<.001	-0.61	0.06	<.001	-0.91	0.05	<.001
	November 2021	-0.39	0.05	<.001	-0.42	0.05	<.001	-0.59	0.05	<.001
	December 2021	-0.18	0.05	<.001	-0.19	0.05	<.001	-0.24	0.04	<.001
	January 2022	Ref	Ref	Ref	Ref	Ref	Ref	Ref	Ref	Ref
	February 2022	-0.16	0.05	<.001	-0.16	0.05	=.001	-0.28	0.05	<.001
	March 2022	-0.23	0.06	<.001	-0.22	0.06	<.001	-0.54	0.05	<.001
	April 2022	-0.65	0.08	<.001	-0.67	0.08	<.001	-1.03	0.05	<.001
	May 2022	-0.46	0.08	<.001	-0.52	0.08	<.001	-0.86	0.06	<.001
	June 2022	-0.64	0.08	<.001	-0.70	0.07	<.001	-0.96	0.05	<.001
	Weekend	-0.06	0.02	<.001	-0.06	0.02	=.01	-0.08	0.02	<.001
	Weekday	Ref	Ref	Ref	Ref	Ref	Ref	Ref	Ref	Ref
	Young adults	0.33	0.31	=.29	0.33	0.31	=.28			
	Middle-aged adults	Ref	Ref	Ref	Ref	Ref	Ref			
	Older adults	-0.63	0.28	=.02	-0.63	0.28	=.02			

	Underweight	0.24	0.30	=.42	0.24	0.30	=.42			
	Healthy weight	Ref	Ref	Ref	Ref	Ref	Ref			
	Overweight	-0.23	0.19	=.22	-0.24	0.19	=.21			
Daily activity (steps)	Intercept	1559.64	3636.14	=.67	10109.31	602.53	<.001	9337.23	348.87	<.001
	$WBGT_{max}$	605.64	272.97	=.03						
	$WBGT_{max}^2$	-11.48	4.97	=.02						
	Daily Precipitation	-33.94	272.97	<.001						
	$Temp_{max}$				-23.34	14.84	=.12			
	Heat stress day ($WBGT_{max}$≥30°C)							-64.41	100.03	=.52
	$WBGT_{max}$<30°C							Ref	Ref	Ref
	Heavy precipitation (≥20mm)							-854.90	262.93	<.001
	Precipitation <20mm							Ref	Ref	Ref
	August 2021	5448.00	271.37	<.001	5137.33	169.43	<.001	5323.41	175.26	<.001
	September 2021	2343.91	272.71	<.001	2254.83	169.80	<.001	2306.46	185.77	<.001
	October 2021	1190.29	265.20	<.001	1188.71	156.99	<.001	1163.19	174.62	<.001
	November 2021	2305.76	226.64	<.001	2577.40	161.48	<.001	2476.49	146.08	<.001
	December 2021	221.07	158.48	=.16	381.78	143.72	=.01	335.13	140.57	=.02
	January 2022	Ref	Ref	Ref	Ref	Ref	Ref	Ref	Ref	Ref
	February 2022	-4.88	172.21	=.098	200.68	155.58	=.20	120.94	146.59	=.41
	March 2022	-284.42	227.17	=.21	4.38	170.16	=.98	-121.49	149.41	=.42
	April 2022	-102.29	250.82	=.68	71.75	182.57	=.69	-37.21	164.06	=.82
	May 2022	-320.65	262.56	=.22	-236.33	176.43	=.18	-318.66	167.74	=.06
	June 2022	1503.16	259.09	<.001	1465.64	161.95	<.001	1513.01	168.24	<.001
	Weekend	-418.29	73.42	<.001	-415.23	73.47	<.001	-417.13	73.47	<.001
	Weekday	Ref	Ref	Ref	Ref	Ref	Ref	Ref	Ref	Ref
	Young adults	1409.37	1393.38	=.31	1418.60	1392.14	=.31	1413.04	1391.71	=.31
	Middle-aged adults	Ref	Ref	Ref	Ref	Ref	Ref	Ref	Ref	Ref

	Older adults	-7463.63	1239.27	<.001	-7451.45	1238.17	<.001	-7448.25	1237.78	<.001
Average nighttime HR (bpm)	Intercept	70.72	2.82	<.001	71.26	3.67	<.001	70.12	1.19	<.001
	HI_{min}	-0.08	0.26	=.75						
	HI_{min}^2	0.00	0.01	=.65						
	Daily Precipitation	-0.04	0.02	=.05	-0.04	0.02	=.06			
	$Temp_{min}$				-0.14	0.01	=.67			
	$Temp_{min}^2$				0.00	0.01	=.56			
	Heat stress night (HI_{min}≥25°C)							-0.27	0.38	=.49
	HI_{min}<25°C							Ref	Ref	Ref
	Heavy precipitation (≥20mm)							-2.01	0.92	=.003
	Precipitation <20mm							Ref	Ref	Ref
	August 2021	-5.75	1.03	<.001	-5.78	0.96	<.001	-5.55	0.73	<.001
	September 2021	-2.57	1.01	<.01	-2.60	0.95	=.01	-2.34	0.71	<.001
	October 2021	-1.65	1.00	=.10	-1.68	0.93	=.07	-1.40	0.71	=.05
	November 2021	-1.48	0.82	=.07	-1.50	0.78	=.06	-1.44	0.70	=.04
	December 2021	-1.26	0.70	=.07	-1.26	0.70	=.07	-1.26	0.69	=.07
	January 2022	Ref	Ref	Ref	Ref	Ref	Ref	Ref	Ref	Ref
	February 2022	-1.60	0.73	=.03	-1.61	0.73	=.03	-1.60	0.70	=.02
	March 2022	-2.96	0.98	<.001	-3.08	0.96	<.001	-2.70	0.72	<.001
	April 2022	-0.96	1.06	=.36	-1.10	1.02	=.28	-0.43	0.74	=.56
	May 2022	-4.13	1.14	<.001	-4.22	1.08	<.001	-3.42	0.78	<.001
	June 2022	-4.35	1.06	<.001	-5.78	1.00	<.001	-3.93	0.76	<.001
	Males	-2.62	1.40	=.07	-2.62	1.40	=.07	-2.62	1.40	=.07
	Females	Ref	Ref	Ref	Ref	Ref	Ref	Ref	Ref	Ref
	Young adults	12.14	4.12	=.01	12.12	4.12	=.01	12.15	4.13	=.01
	Middle-aged adults	Ref	Ref	Ref	Ref	Ref	Ref	Ref	Ref	Ref
	Older adults	-0.81	2.30	=.73	-0.81	2.30	=.73	-0.78	2.30	=.74

	Underweight	-4.78	2.65	=.08	-4.78	2.65	=.08	-4.76	2.65	=.08
	Healthy weight	Ref	Ref	Ref	Ref	Ref	Ref	Ref	Ref	Ref
	Overweight	3.50	1.80	=.06	3.51	1.80	=.06	3.47	1.80	=.06

Table S2. Fully adjusted models for the dataset with a complete case cutoff of 50%: Model 1 (WBGT or HI and total daily precipitation as predictors), Model 2 (temperature and total daily precipitation as predictors) and Model 3 (binary variable heat stress day/night as predictor) for daily activity and sleep duration. For sleep duration as the outcome variable Model 1 had an AIC of 56290, Model 2 an AIC of 56291 and model 3 had an AIC of 56461. For the outcome steps Model 1 had an AIC of 374919, Model 2 had an AIC of 374943 and Model 3 had an AIC of 374944.

		Model 1			Model 2			Model 3		
Independent	Predictor	Estimate	SE	p-value	Estimate	SE	p-value	Estimate	SE	p-value
Sleep duration (h)	Intercept	7.96	0.12	<.001	8.09	0.13	<.001	7.40	0.09	<.001
	HI_{min}	-0.04	0.00	<.001						
	Daily Precipitation	0.01	0.00	<.001	0.01	0.00	<.001			
	$Temp_{min}$				-0.04	0.01	<.001			
	Heat stress night (HI_{min}≥25°C)							-0.24	0.04	<.001
	HI_{min}<25°C							Ref	Ref	Ref
	August 2021	-0.29	0.08	<.001	-0.34	0.08	<.001	-0.54	0.06	<.001
	September 2021	-0.67	0.08	<.001	-0.71	0.08	<.001	-0.97	0.06	<.001
	October 2021	-0.50	0.08	<.001	-0.55	0.07	<.001	-0.83	0.06	<.001
	November 2021	-0.36	0.06	<.001	-0.39	0.06	<.001	-0.56	0.05	<.001
	December 2021	-0.14	0.05	<.001	-0.15	0.05	<.001	-0.20	0.05	<.001
	January 2022	Ref	Ref	Ref	Ref	Ref	Ref	Ref	Ref	Ref
	February 2022	-0.14	0.05	=.01	-0.14	0.05	=.001	-0.25	0.05	<.001
	March 2022	-0.23	0.07	<.001	-0.22	0.07	<.001	-0.52	0.05	<.001
	April 2022	-0.58	0.09	<.001	-0.60	0.08	<.001	-0.93	0.06	<.001
	May 2022	-0.42	0.09	<.001	-0.47	0.09	<.001	-0.79	0.06	<.001
	June 2022	-0.61	0.08	<.001	-0.67	0.08	<.001	-0.91	0.06	<.001
	Weekend	-0.08	0.03	<.001	-0.08	0.03	=.01	-0.09	0.03	<.001
	Weekday	Ref	Ref	Ref	Ref	Ref	Ref	Ref	Ref	Ref

	Young adults	0.57	0.30	=.06	0.57	0.30	=.06	0.59	0.30	=.05
	Middle-aged adults	Ref	Ref	Ref	Ref	Ref	Ref	Ref	Ref	Ref
	Older adults	-1.94	0.67	<.001	-0.57	0.30	=.01	-2.13	0.65	<.001
	Underweight	0.11	0.38	=.77	0.11	0.38	=.78			
	Healthy weight	Ref	Ref	Ref	Ref	Ref	Ref			
	Overweight	-0.23	0.20	=.26	-0.23	0.20	=.25			
Daily activity (steps)	Intercept	2772.31	4007.31	=.49	10270.84	670.34	<.001	9319.30	408.73	<.001
	$WBGT_{max}$	518.00	300.57	=.08						
	$WBGT_{max}^2$	-10.03	5.47	=.07						
	Daily Precipitation	-30.83	5.77	<.001						
	$Temp_{max}$				-28.77	16.06	=.07			
	Heat stress day ($WBGT_{max}$≥30°C)							-58.44	109.72	=.59
	$WBGT_{max}$<30°C							Ref	Ref	Ref
	Heavy precipitation (≥20mm)							-541.96	293.30	=.06
	Precipitation <20mm							Ref	Ref	Ref
	August 2021	5597.90	301.69	<.001	5218.61	189.64	<.001	5366.02	196.15	<.001
	September 2021	2179.47	302.32	<.001	2004.78	189.11	<.001	2045.41	206.27	<.001
	October 2021	1278.28	294.81	<.001	1203.46	176.26	<.001	1152.88	195.74	<.001
	November 2021	2345.43	252.56	<.001	2566.02	181.08	<.001	2438.16	165.07	<.001
	December 2021	148.99	176.96	=.40	288.70	160.60	=.07	231.20	157.36	=.14
	January 2022	Ref	Ref	Ref	Ref	Ref	Ref	Ref	Ref	Ref
	February 2022	117.15	188.42	=.53	302.67	169.13	=.07	203.54	159.46	=.20
	March 2022	-260.71	248.71	=.29	-19.94	183.22	=.91	-176.68	160.34	=.27
	April 2022	-33.57	273.99	=.90	88.98	196.10	=.65	-63.98	176.38	=.72
	May 2022	-341.31	286.17	=.23	-315.36	188.48	=.09	-435.41	179.42	=.02
	June 2022	1580.39	283.11	<.001	1472.72	174.04	<.001	1490.24	181.15	<.001
	Weekend	-488.41	81.10	<.001	-487.38	81.15	<.001	-488.76	81.15	<.001

	Weekday	Ref	Ref	Ref	Ref	Ref	Ref	Ref	Ref	Ref
	Young adults	1814.55	1646.40	=.27	1825.72	1643.99	=.27	1823.17	1643.97	=.27
	Middle-aged adults	Ref	Ref	Ref	Ref	Ref	Ref	Ref	Ref	Ref
	Older adults	-7383.98	1647.47	<.001	-7375.28	1645.06	<.001	-7378.31	1645.04	<.001
